# Efficacy and safety of Shenmai injection for acute ischemic stroke: a systematic review and meta-analysis

**DOI:** 10.3389/fphar.2024.1394936

**Published:** 2024-06-04

**Authors:** Shuai Zhao, Tianye Sun, Mi Zhang, Mingyuan Yan, Kaiyue Wang, Lili Li, Jinmin Liu

**Affiliations:** ^1^ Beijing University of Chinese Medicine, Beijing, China; ^2^ Dongfang Hospital, Beijing University of Chinese Medicine, Beijing, China

**Keywords:** acute ischemic stroke, Shenmai injection, efficacy, safety, randomized controlled trials, systematic review, meta-analysist

## Abstract

**Background:**

Ischemic stroke is a serious and sudden cerebrovascular condition that significantly affects individual’s health and imposes a substantial economic burden on medical management. Despite its widespread use in China, there is still a lack of reliable evidence regarding the efficacy of Shenmai injection (SMI) in acute ischemic stroke (AIS). We aimed to comprehensively assess the effectiveness and safety of SMI in treating AIS through a systematic review and meta-analysis.

**Methods:**

Randomized controlled studies (RCTs) investigating the efficacy of SMI in treating AIS were searched for in eight databases from the inception of each database till January 2024. We utilized the ROB 2.0 to assess the risk of bias. A meta-analysis was conducted using Review Manager 5.4, while sensitivity analyses and publication bias assessments were conducted using Stata 16.1.

**Results:**

A total of 17 studies involving 1,603 AIS patients were included in our meta-analysis. Our results showed that SMI plus conventional treatments (CTs) was more effective than CTs alone in improving the total effective rate (RR 1.22, 95% CI: 1.14 to 1.30, *p* < 0.00001), the Barthel index (BI) (MD 12.18, 95% CI: 10.30 to 14.06, *p* < 0.00001), and reducing the National Institute of Health Stroke Scale Score (NIHSS) score (MD -3.05, 95% CI: 3.85 to −2.24, *p* < 0.00001) and Modified Rankin Scale (mRS) (MD -0.68, 95% CI: 0.86 to-0.49, *p* < 0.00001). In addition, SMI combination therapy was better than CTs alone in decreasing the levels of IL-6, IL-18, and hs-CRP. SMI therapy also enhanced the cerebral hemorheology of patients by reducing levels of fibrinogen and plasma viscosity. However, there was no significant difference in the incidence of adverse events, including elevated transaminase, rash, nausea, bleeding, urticaria, headache, vomiting, chest tightness, and facial flushes. Moreover, no serious adverse effects or life-threatening events were reported.

**Conclusion:**

Our study shows that combining SMI with CTs effectively enhances the neurological function of patients with acute cerebral infarction. However, our findings should be interpreted considering the significant heterogeneity and suboptimal quality of the analyzed trials.

**Systematic review registration:**

https://www.crd.york.ac.uk/prospero/display_record.php?ID=CRD42024504675, Identifier PROSPERO, CRD42024504675

## 1 Introduction

Acute ischemic stroke (AIS) refers to sudden focal ischemia of the brain tissue lasting more than 24 h and causing neurological dysfunction (Mendelson and Prabjakaran, 2021). Ischemic stroke, accounting for 87% of all stroke occurrences, is a major cause of mortality and disability globally, and the clinical prognosis of AIS is poor (Liu et al., 2020; Tsao et al., 2022). Currently, stroke is the leading cause of death in China, with an annual increase of 2.5 million cases and 1.6 million deaths (Jia et al., 2022).

Stroke can result in various complications, including neurological abnormalities, infections, movement impairment, thrombosis, and emotional issues (Lin et al., 2022). Among these complications, recurrent stroke is regarded as the most serious; patients with repeated stroke frequently become locked in a vicious cycle, resulting in fast functional decline (Saengsuwan et al., 2017). Furthermore, a study conducted in rural China found that the age-standardized risk of recurrent stroke within 5 years was 22.5% (24.0% for men and 20.2% for women) (Han et al., 2020). AIS is a significant health issue that imposes a substantial economic burden on families and society.

The main objective of AIS treatment is to reopen the arteries and restore tissue blood flow (Sapir et al., 2021). Recent advances in AIS management, including intravenous thrombolysis (IVT) and endovascular thrombectomy (EVT), have significantly improved post-stroke survival rates (Powers et al., 2019). However, the use of IVTs and endovascular thrombectomy is limited due to the occurrence of hemorrhagic transformation and reperfusion injury after recanalization (Zhang et al., 2021; Bo et al., 2022). Furthermore, it is essential to consider the constraints imposed by the narrow therapeutic window (Dong et al., 2021). Hence, identifying effective therapies for AIS is critical.

Shenmai injection (SMI) is a well-known botanical drug injection extracted from *Panax ginseng C.A. Mey.* [Araliaceae; Radix ginseng rubra] and *Ophiopogon japonicus* (Thunb.) Ker Gawl. [Asparagaceae; Ophiopogonis Radix], which was included in the National Essential Drugs List (2018 Edition) in China. Regarding the preparation thereof, the pharmaceutical factory acquired 100 g each of Radix ginseng rubra and Ophiopogonis Radix. These were heated and refluxed with 90% ethanol for extraction, then subjected to reduced pressure to yield 0.3–0.4 g of raw medicine per 1 mL. Following filtration with activated carbon and dilution with water, 5 g of polysorbate 80 was added. Injection water was added to reach a volume of 1,000 mL, and the pH was adjusted to 5.0–6.5 using 10% NaOH. Lastly, the mixture underwent filtration, sealing, and sterilization to produce SMI. In accordance with the drug standards set by the China Food and Drug Administration (China Food and Drug Administration, 2010), the concentration of Ginsenoside Re, an active metabolite traditionally employed to ensure the quality of SMI, should not be lower than 0.8 g/L. The details of SMI, including the composition, source, actions, quality control, etc., are shown in Supplementary Table S2, and the principal chemical constituents of SMI under fingerprint methods are summarized in Supplementary Table S3. Research has shown that SMI can successfully mitigate the damage caused by ischemia-reperfusion, enhance cerebral blood flow, and decrease the extent of cerebral infarction (Zuo et al., 2015). In addition, pharmacological studies have proven that SMI exerts neuro-protective effects by preserving blood-brain barrier functional integrity, reducing oxidative stress, and inhibiting neuronal apoptosis (Dang et al., 2017; Xiao et al., 2019; Xu et al., 2019). Moreover, SMI has demonstrated the capability to lower the levels of NO, NOS, and MDA in brain tissue while increasing SOD activity to enhance the removal of oxygen radicals (Zhao et al., 2012). Clinical trials have revealed that SMI improves neurological function, cerebral hemodynamics, and reduces inflammatory factor levels in patients with AIS (Sun, 2020).

A previous meta-analysis studied SMI in the treatment of AIS (Liu et al., 2017), but with certain restrictions. Firstly, the inclusion of other TCM interventions in the literature considered in this study could have affected the precision of the findings. Secondly, the study exhibited significant heterogeneity and did not conduct subgroup and sensitivity analyses to assess stability. Additionally, the assessment of the effectiveness and safety of SMI was not comprehensive due to the restricted range of outcome measures. Due to these limitations, the results are less reliable and less applicable to clinical settings.

We, therefore, aimed to conduct a subgroup analysis to determine the influence of SMI on the efficacy and safety of AIS, and use standardized criteria to improve the accuracy of the pharmacological efficacy evaluation. Moreover, serum inflammatory markers and coagulation indices were included as important indicators to assess effectiveness. The GRADE system was utilized to evaluate evidence quality, offering clinicians a reliable and objective reference for SMI.

## 2 Materials and methods

This systematic review and meta-analysis follows the Preferred Reporting Items for Systematic Reviews and Meta-Analyses Statement. The study protocol was registered with PROSPERO (registration number: CRD42024504675).

### 2.1 Search strategy

We searched the PubMed, Embase, Web of Science, Cochrane Library, China National Knowledge Infrastructure, Chinese Sci-tech Periodical Database (VIP), Chinese Biological Medicine Database (Sinomed), and the Chinese Wan Fang Database (Wan fang) from the inception of the database to February 2024. The search strategies for each database are depicted in Supplementary Material S1.

### 2.2 Selection criterion

#### 2.2.1 Types of studies

The study comprised randomized controlled trials (RCTs) with participants meeting the diagnostic criteria for AIS.

#### 2.2.2 Types of interventions

Conventional treatments (CTs) were used in the control group (antiplatelet, anticoagulation, improvement of cerebral circulation, nerve nutrition, radical scavenging, defibrase, and statins) (Chinese Society of Neurology and Chinese Stroke Society, 2018). Patients in the experimental group were administered SMI in conjunction with conventional treatments. Studies with the other TCM treatments were excluded.

#### 2.2.3 Outcome measures

The primary outcome was total effective rate, which is determined by calculating the decrease in the National Institute of Health Stroke Scale Score (NIHSS); we defined the total effective rate as the ratio of patients whose NIHSS score reduced by >18%. Secondary outcomes included the NIHSS, Barthel Index (BI), modified Rankin Scale (mRS), and other related scales, interleukin-6 (IL-6), interleukin-18 (IL-18), C-reactive protein (hs-CRP), fibrinogen levels, plasma viscosity, hematocrit, and adverse events.

### 2.3 Exclusion criteria

The following studies were excluded from our analysis: 1) case reports, experiments, reviews, and conference papers; 2) studies that included other forms of traditional Chinese medicine, such as herbal remedies, acupuncture, massage, and scraping; 3) studies with three arms; 4) studies with data that was incomplete or unable to be extracted; and 5) studies with patients treated with IVTs or EVTs.

### 2.4 Study selection and data extraction

EndNote X9.1 was used to organize the studies for analysis. After removing duplicates, two reviewers (ZS and STY) independently evaluated the titles and abstracts to exclude non-compliant papers. Then, to find other studies to include, the whole texts of the remaining studies were downloaded and thoroughly reviewed. For each included study, two writers separately retrieved information on the authors, publication date, gender, age, sample size, intervention, the injection dosage of SMI, length of treatment, and outcome metrics. A third author (ZM) arbitrated any disputes between the two reviewers.

### 2.5 Risk of bias

Two reviewers (ZS and STY) independently assessed and cross-checked the risk of bias for eligible RCTs using the Cochrane risk of bias tool 2.0 (RoB 2.0). Six domains were evaluated: 1) randomization process, 2) deviations from the intended interventions, 3) missing outcome data, 4) measurement of the outcome, 5) selection of the reported outcome, and 6) overall bias. The trials were all categorized as having a low, uncertain, or high risk of bias. Disputes were resolved through consultation with a third researcher (ZM) or through mutual negotiation.

### 2.6 Data analysis

A meta-analysis was performed using the RevMan 5.4 or Stata 16.1 software. Dichotomous results were analyzed using risk ratio (RR) and 95% confidence intervals (CIs), whereas continuous results were examined using mean difference (MD) and 95% CIs. A *p*-value of <0.05 was deemed to show statistical significance for the overall impact.

To explore potential sources of heterogeneity, we conducted subgroup analyses, considering the dosage of SMI (20 mL, 40 mL, 50 mL, 60 mL, 60–100 mL), intervention duration (10 D, 2 W, 3 W, 4 W). Heterogeneity was evaluated using the I^2^ value, and values exceeding 50% were considered indicative of significant statistical heterogeneity (Sun et al., 2010). We selected a random effect model when there was significant heterogeneity. Otherwise, a fixed effects model was adopted. Heterogeneity was explained by subgroup or sensitivity analyses if the random-effects model was applicable. The sensitivity analyses were conducted by systematically excluding individual studies one at a time to assess the impact of their heterogeneity on the combined results of the meta-analyses. We evaluated publication bias by creating funnel plots and conducting both the Egger’s and Harbord tests. When the *p*-value of Egger’s tests was <0.05, the trim-and-fill calculation was used to evaluate the effect of publication bias.

## 3 Results

### 3.1 Study selection

**FIGURE 1:**
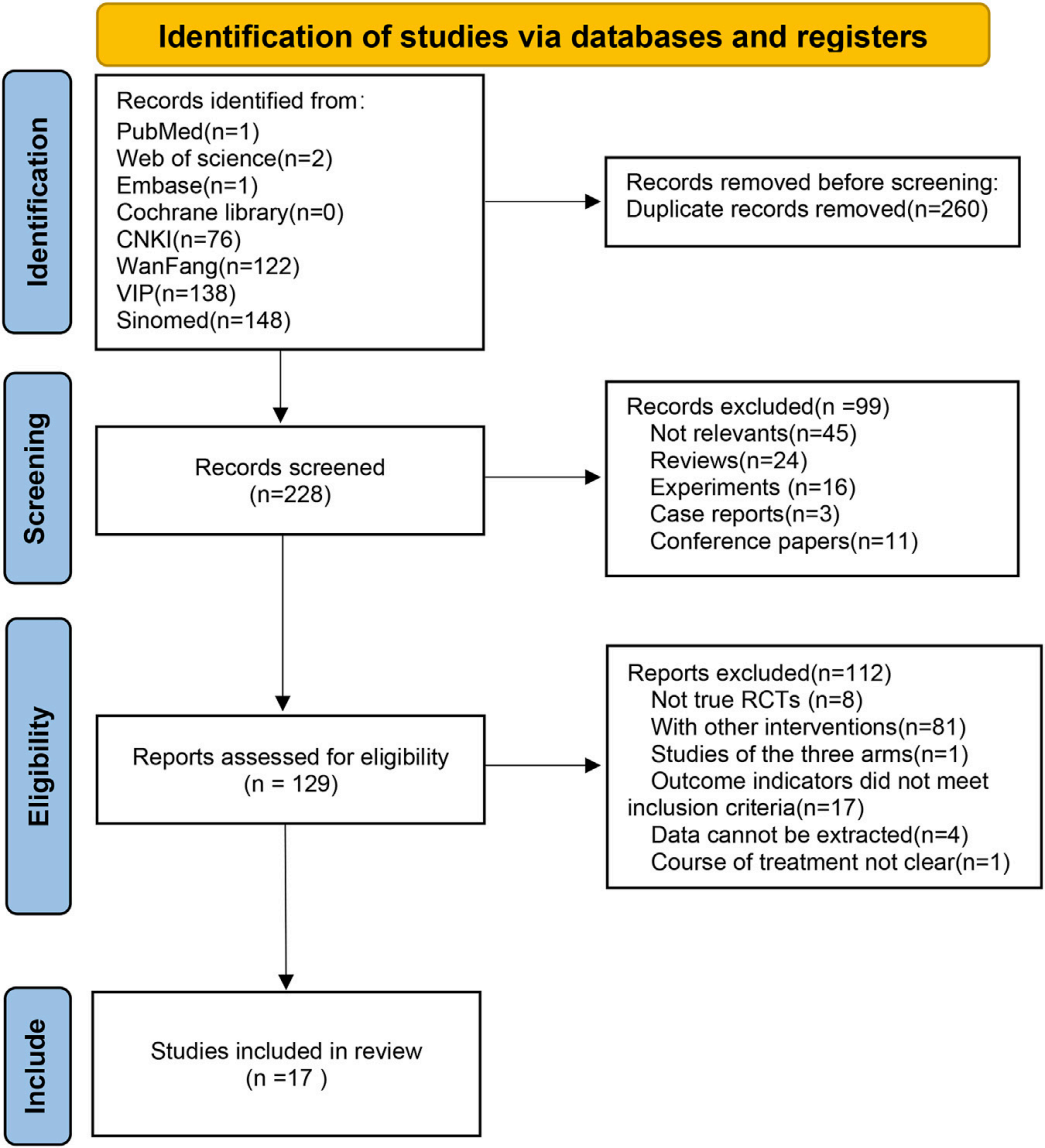
PRISMA flow chart for study selection.

Overall, we initially gathered 488 studies showing relevance from the eight databases. After eliminating 260 duplicates, the titles and abstracts of the remaining 228 studies were examined. Among them, 99 studies were removed due to not meeting the inclusion criteria. A total of 112 articles were further excluded because they were not true RCTs (*n* = 8), were other interventions (*n* = 81), had outcome indicators that did not meet the inclusion criteria (*n* = 17), had data that could not be extracted (*n* = 4), had unclear courses of treatment (*n* = 1), and were studies with three arms (*n* = 1). Finally, 17 studies (Tang, 2011; Lu and Yang, 2013; Wang et al., 2013; Wang, 2014; Liu et al., 2016; Chen and Liu, 2018; Han, 2018; Hu, 2018; Liang et al., 2018; Wu et al., 2018; Zhang et al., 2018; Chen, 2019; Li and Chen, 2019; Liu et al., 2020; Sun, 2020; Li and Dai, 2022; Wu, 2022) were included for the final meta-analysis (Figure 1).

### 3.2 Study characteristics

A total of 1,603 individuals were enrolled, of which 804 were in the group that received SMI combined with CTs and 799 were in the group that received CTs alone. All the included studies were RCTs conducted in China, with a publication time range from 2011 to 2022. The participants had an average age ranging from 56.9 to 78.9 years old, with onset within 14 days. The dosage of SMI treatment exhibited variation, with a minimum of 20 mL (Li and Dai, 2022) and a maximum of 100 mL (Han, 2018). The duration of treatment ranged from a minimum of 10 days (Liu et al., 2016) to a maximum of 4 weeks (Han, 2018; Liu et al., 2020; Li and Dai, 2022). The administration of SMI to experimental groups was conducted by intravenous route in all studies, with a frequency of once-daily treatment. A summary of the fundamental characteristics and specific information of the studies included in the analysis is presented in Table 1.

**TABLE 1:** The characteristics of included studies.

Study	Sample size (T/C)	Age (year)		Gender (M/F)		Course of diease (hour/day)		Interventions		Duration	Outcomes
		T	C	T	C	T	C	T	C		
Wu. (2022)	30/30	64.12 ± 2.28	64.24 ± 2.31	17/13	16/14	12.21 ± 0.45 h	12.38 ± 0.48 h	SMI 50 mL qd, ivgtt + CTs	CTs	2 weeks	①②③④⑤⑥⑦
Li and Dai. (2022)	35/35	61.24 ± 4.57	61.32 ± 4.61	20/15	19/16	3.28 ± 0.66 h	3.33 ± 0.78 h	SMI 20 mL qd, ivgtt + CTs	CTs	4 weeks	②⑤⑥
Sun. (2020)	37/37	65.7 ± 6.2	66.1 ± 6.1	23/14	23/14	23.80 ± 8.00 h	24.21 ± 9.78 h	SMI 50 mL qd, ivgtt + CTs	CTs	2 weeks	②⑤⑦
Liu et al. (2016)	60/60	65.11 ± 9.74	66.33 ± 5.09	33/27	37/23	0–2 h		SMI 50 mL qd, ivgtt + CTs	CTs	10 days	②⑤
Chen. (2019)	60/60	69.21 ± 3.47	68.38 ± 3.57	46/14	45/15	NR		SMI 50 mL qd, ivgtt + CTs	CTs	2 weeks	①②③④
Hu. (2018)	57/57	60.11 ± 10.24	61.24 ± 10.43	26/31	28/29	NR		SMI 40 mL qd, ivgtt + CTs	CTs	2 weeks	②⑦
Wang. (2014)	28/28	62.5 ± 8.2	59.6 ± 8.5	16/12	18/10	0–24 h		SMI 40 mL qd, ivgtt + CTs	CTs	2 weeks	①②③⑤
Tang. (2011)	35/30	51–79		33/32		0–48		SMI 60 mL qd, ivgtt + CTs	CTs	2 weeks	⑤⑥
Han. (2018)	54/54	65.18 ± 6.72	64.57 ± 6.85	33/21	32/22	6–24 h		SMI 60–100 mL qd, ivgtt + CTs	CTs	4 weeks	⑤
Chen and Liu. (2018)	47/47	78. 47 ± 5.62	78. 91 ± 6.13	27/20	28/19	20.38 ± 4.35 h	19.93 ± 4.18 h	SMI 60 mL qd, ivgtt + CTs	CTs	2 weeks	①③⑥
Lu and Yang. (2013)	32/32	57.5 ± 8.2	58.4 ± 7.8	17/15	18/14	0–24 h		SMI 40 mL qd, ivgtt + CTs	CTs	2 weeks	①②⑤
Liu et al. (2020)	59/59	63.05 ± 5.14	62.54 ± 5.29	37/22	34/25	13.23 ± 3.45 h	13.61 ± 3.22 h	SMI 50 mL qd, ivgtt + CTs	CTs	4 weeks	①②⑤⑦
Wang et al. (2013)	30/30	38–75	41–73	19/11	17/13	0–48 h		SMI 60 mL qd, ivgtt + CTs	CTs	2 weeks	⑤
Li and Chen. (2019)	70/70	73.5 ± 3.4	73.1 ± 3.9	38/32	35/35	32.9 ± 3.1 h	32.3 ± 2.5 h	SMI 50 mL qd, ivgtt + CTs	CTs	2 weeks	②⑦
Liang et al. (2018)	40/40	58.80 ± 14.87	56.90 ± 15.24	27/13	25/15	0-5d		SMI 50 mL qd, ivgtt + CTs	CTs	3 weeks	①②
Zhang et al. (2018)	70/70	61.3 ± 4.6	62.1 ± 5.3	39/31	37/33	0–48 h		SMI 50 mL qd, ivgtt + CTs	CTs	2 weeks	①②③④⑤⑥⑦
Wu et al. (2018)	60/60	64.3 ± 9.4	65.8 ± 9.5	33/27	34/26	0-14d		SMI 40 mL qd, ivgtt + CTs	CTs	2 weeks	②

①Total effective rate ②National Institute of Health stroke scale (NIHSS) score③Barthel index (BI) ④Modified Rankin Scale (mRS) ⑤Levels of Inflammatory Factors ⑥Levels of Hemorheology ⑦Adverse events.

### 3.3 Risk of bias assessment

**FIGURE 2:**
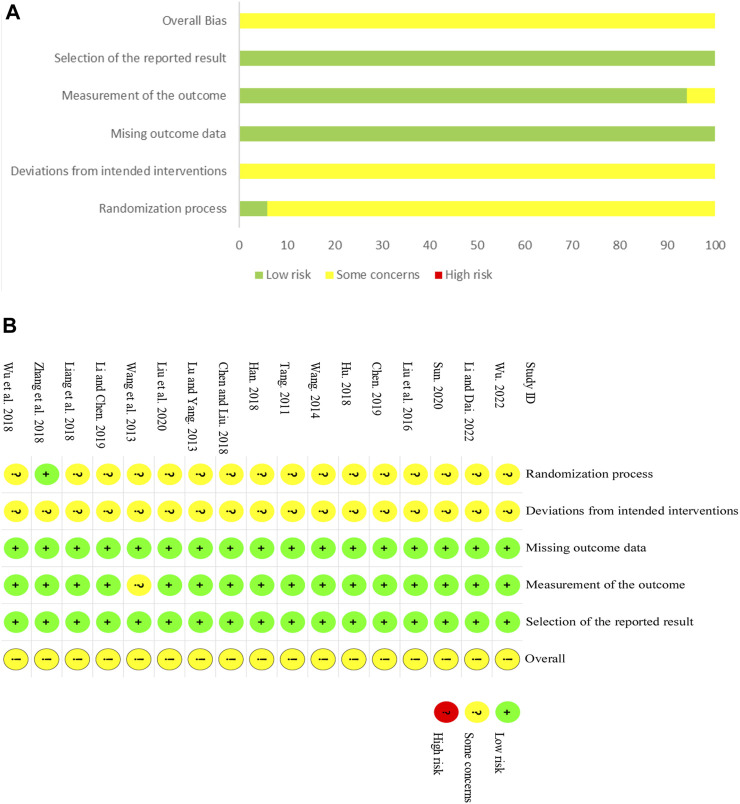
Risk of bias. **(A)** Risk of bias summary. **(B)** Risk of bias graph.

In general, most of the analyzed trials demonstrated low-to-moderate levels of quality. Among the analyzed studies, 11 (Lu and Yang, 2013; Wang, 2014; Chen and Liu, 2018; Han, 2018; Hu, 2018; Liang et al., 2018; Wu et al., 2018; Li and Chen, 2019; Liu et al., 2020; Sun, 2020; Li and Dai, 2022) were randomly assigned using the random number table method, one (Chen, 2019) used the lottery method, and another (Zhang et al., 2018) employed the randomized block design method. The methodology for random grouping was not explicated in detail in the remaining experiments. Furthermore, the trials included no information about participant blinding, outcome assessment, or allocation concealment. We, therefore, rated the bias risk as uncertain. All 17 articles included in the study contained comprehensive data and did not have any selectively reported findings. Additional biases were categorized as unclear due to inadequate information. Figures 2A, B display a summary and graph of the ROB assessment.

### 3.4 Findings from meta-analyses

#### 3.4.1 Primary efficacy outcome

##### 3.4.1.1 Total effective rate

**FIGURE 3:**
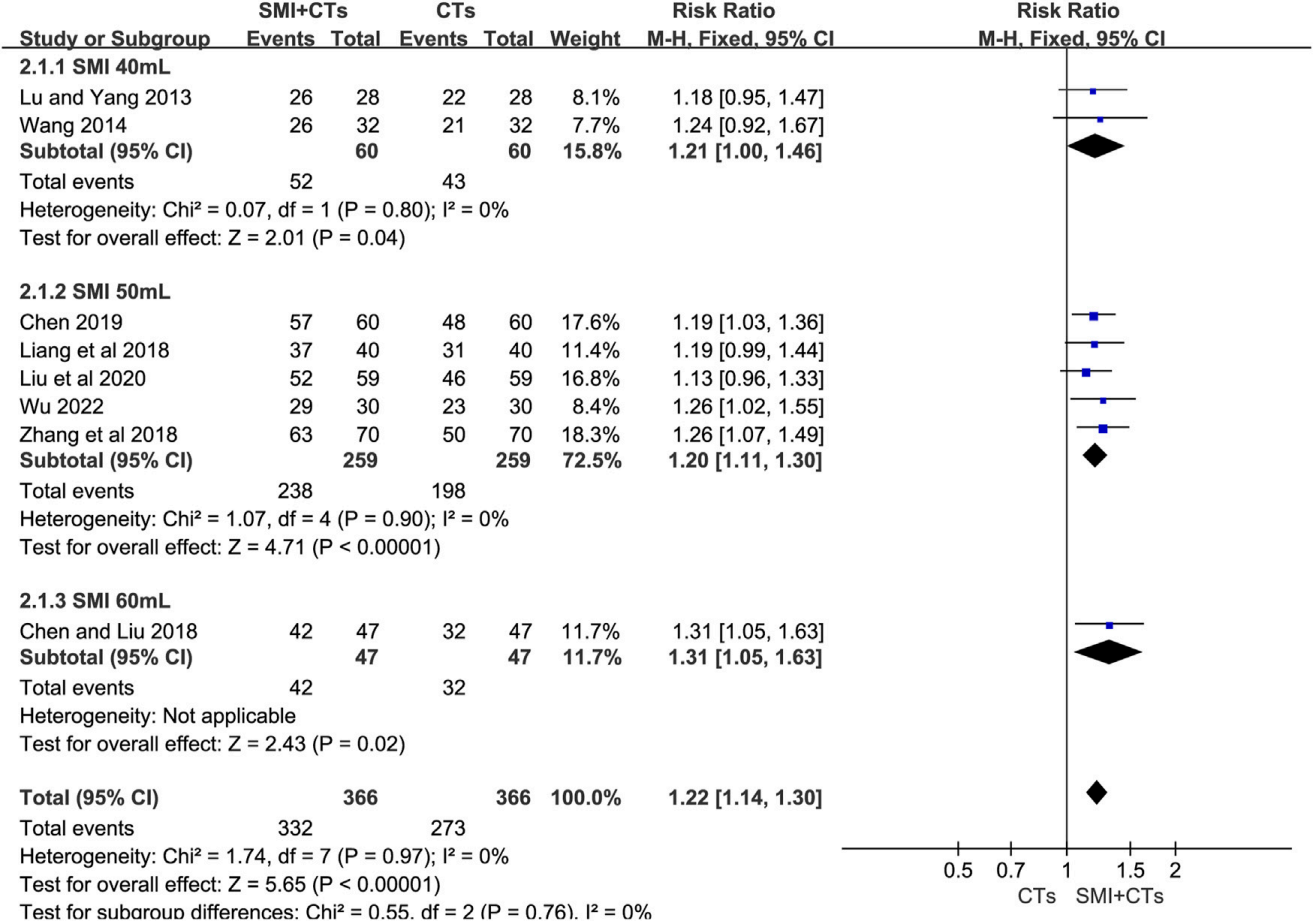
Forest plot for total effective rate.

Eight studies with a total of 732 patients reported the total effective rate, including 366 patients in the SMI combining CTs group and 366 in the CTs alone group (Lu and Yang, 2013; Wang, 2014; Chen and Liu, 2018; Liang et al., 2018; Zhang et al., 2018; Chen, 2019; Liu et al., 2020; Wu, 2022). The meta-analysis findings indicated that the combination of SMI with CTs significantly enhanced the total effective rate compared to CTs alone (RR 1.22, 95% CI: 1.14 to 1.30, *p* < 0.00001, Figure 3). A subgroup analysis was conducted based on the SMI dosage. The total effective rate was significantly higher in groups combining CTs with 60 mL of SMI (RR 1.31, 95% CI: 1.05 to 1.63, *p* = 0.02, Figure 3), 50 mL of SMI (RR 1.20, 95% CI: 1.11 to 1.30, *p* < 0.00001, Figure 3), and 40 mL of SMI (RR 1.21, 95% CI: 1.00 to 1.46, *p* = 0.04, Figure 3) compared to the control group. Furthermore, the sensitivity analysis confirmed the stability of the findings obtained from the meta-analysis (Supplementary Figure S1).

#### 3.4.2 Secondary outcomes

##### 3.4.2.1 NIHSS score

**FIGURE 4:**
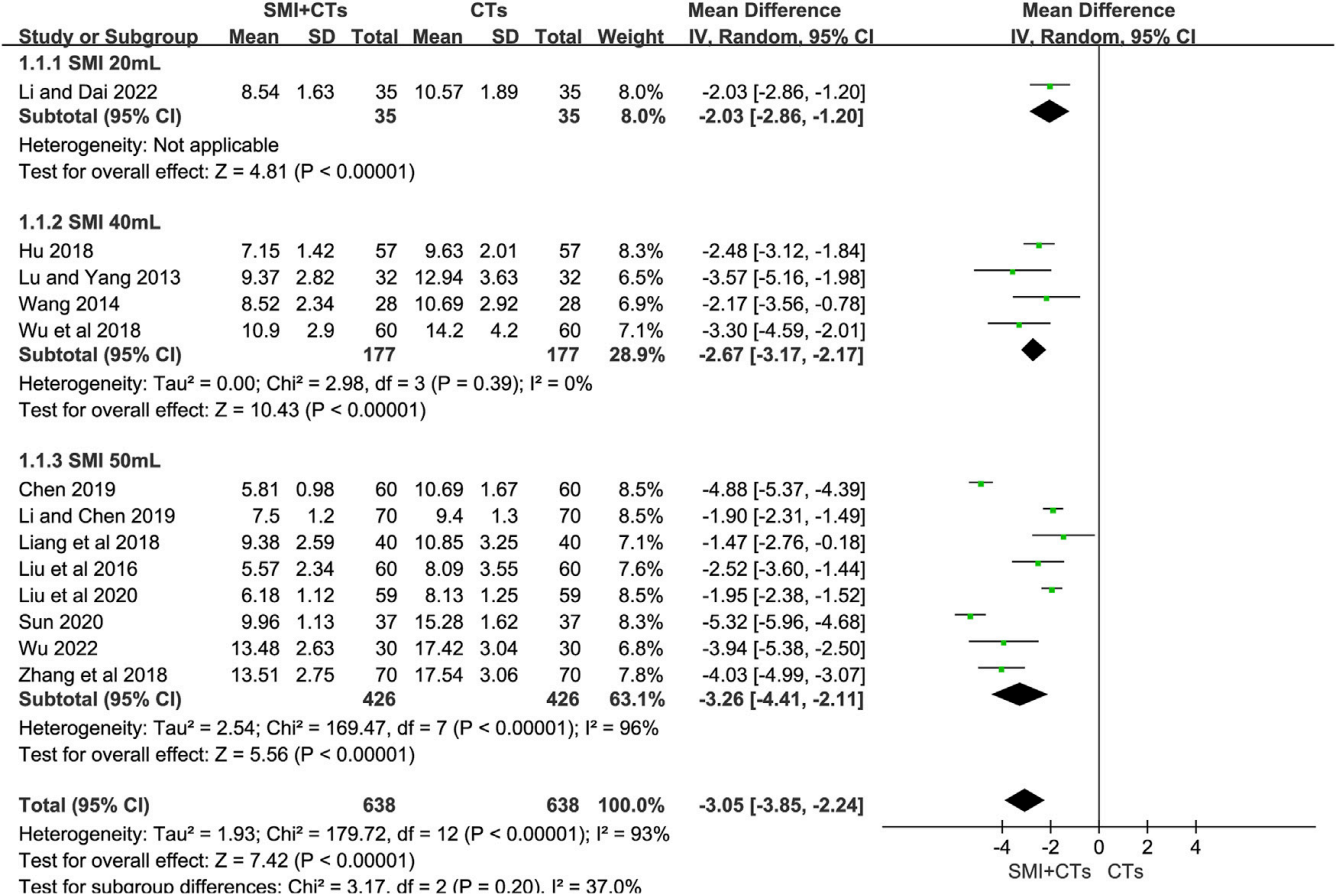
Forest plot for NIHSS score.

The NIHSS score serves as a crucial indicator to assess neurological deficits in patients with AIS. Twelve studies (Lu and Yang, 2013; Wang, 2014; Liu et al., 2016; Hu, 2018; Liang et al., 2018; Wu et al., 2018; Chen, 2019; Li and Chen, 2019; Liu et al., 2020; Sun, 2020; Li and Dai, 2022; Wu, 2022) involving 1,276 patients assessed the changes in NIHSS score. The meta-analysis results showed that combining SMI with CTs led to a significant reduction in the NIHSS score compared to using CTs alone (MD -3.05, 95% CI: 3.85 to −2.24, *p* < 0.00001, Figure 4). A subgroup analysis based on the dosage of SMI revealed significantly lower NIHSS score in groups combining CTs with 50 mL (MD -3.26, 95% CI: 4.41 to −2.11, *p* < 0.00001, I^2^ = 96%, Figure 4), 40 mL (MD -2.67, 95% CI: 3.17 to −2.17, *p* < 0.00001, I^2^ = 0%, Figure 4), and 20 mL (MD -2.03, 95% CI: 2.86 to −1.20, *p* < 0.00001, Figure 4) of SMI compared to CTs alone.

Due to high heterogeneity, the random-effects model was employed (*p* < 0.0001, I^2^ = 93%). A subgroup analysis indicated that treatment dosage might significantly influence heterogeneity, although it may not account for all sources thereof. Egger’s test (*p* = 0.053) suggested no significant publication bias in our analysis (Supplementary Figure S2). Furthermore, a sensitivity analysis was performed to validate the robustness of the meta-analytic results (Supplementary Figure S1).

##### 3.4.2.2 BI

**FIGURE 5:**
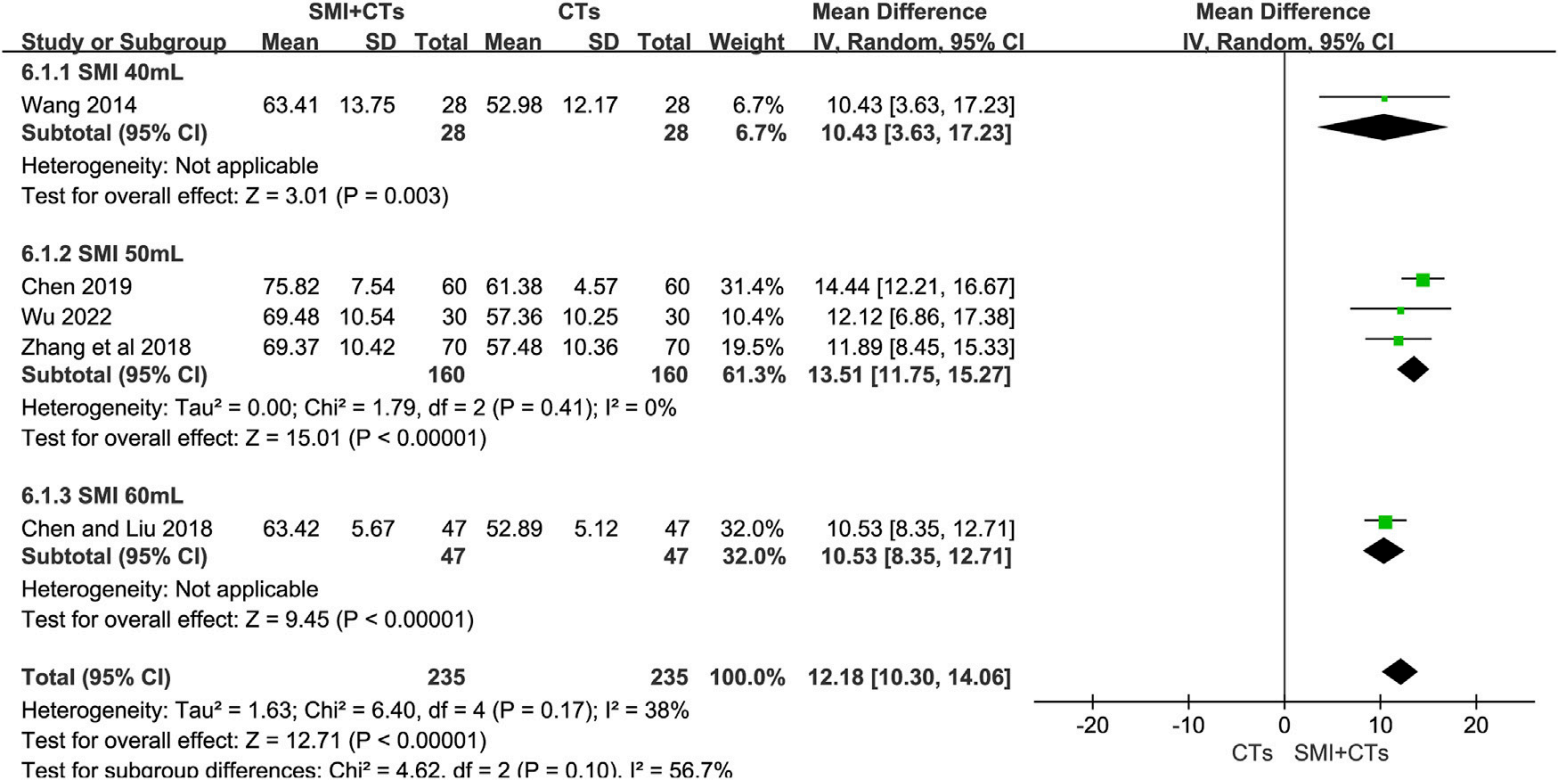
Forest plot for BI.

Five studies (Wang, 2014; Chen and Liu, 2018; Zhang et al., 2018; Chen, 2019; Wu, 2022) involving 470 patients assessed the changes in BI. The meta-analysis results showed that combining SMI with CTs considerably improved the BI compared to using CTs alone (MD 12.18, 95% CI: 10.30 to 14.06, *p* < 0.00001, I^2^ = 38%, Figure 5). A SMI dosage-based subgroup analysis was carried out. The BI was significantly higher in groups combining CTs with 60 mL of SMI (MD 10.53, 95% CI: 8.35 to 12.71, *p* < 0.00001, Figure 5), 50 mL of SMI (MD 13.51, 95% CI: 11.75 to 15.27, *p* < 0.00001, I^2^ = 0%, Figure 5), and 40 mL of SMI (MD 10.43, 95% CI: 3.63 to 17.23, *p* = 0.003, Figure 5) compared to the control group. The results of Egger’s test (*p* = 0.754) indicated that there was no substantial publication bias in our study (Supplementary Figure S2). A sensitivity study was conducted to verify the reliability of the meta-analytic findings (Supplementary Figure S1).

##### 3.4.2.3 Modified rankin scale (mRS)

**FIGURE 6:**
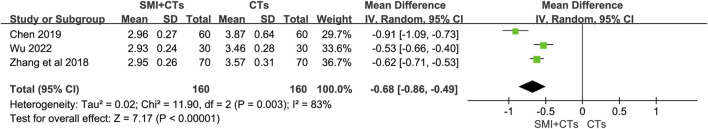
Forest plot for mRS.

The significant heterogeneity that was found (*p* < 0.0001, I^2^ = 83%) led to the selection of the random-effects model. Three studies (Zhang et al., 2018; Chen, 2019; Wu, 2022) involving 320 patients assessed the changes in mRS. The meta-analysis revealed that combining SMI with CTs significantly reduced the mRS compared to CTs alone (MD -0.68, 95% CI: 0.86 to −0.49, *p* < 0.00001, Figure 6). Egger’s test (*p* = 0.567) indicated no significant publication bias in our study (Supplementary Figure S2). Nonetheless, the reliability of the meta-analysis results is compromised by the limited sample size and high heterogeneity.

##### 3.4.2.4 Levels of inflammatory factors

**FIGURE 7:**
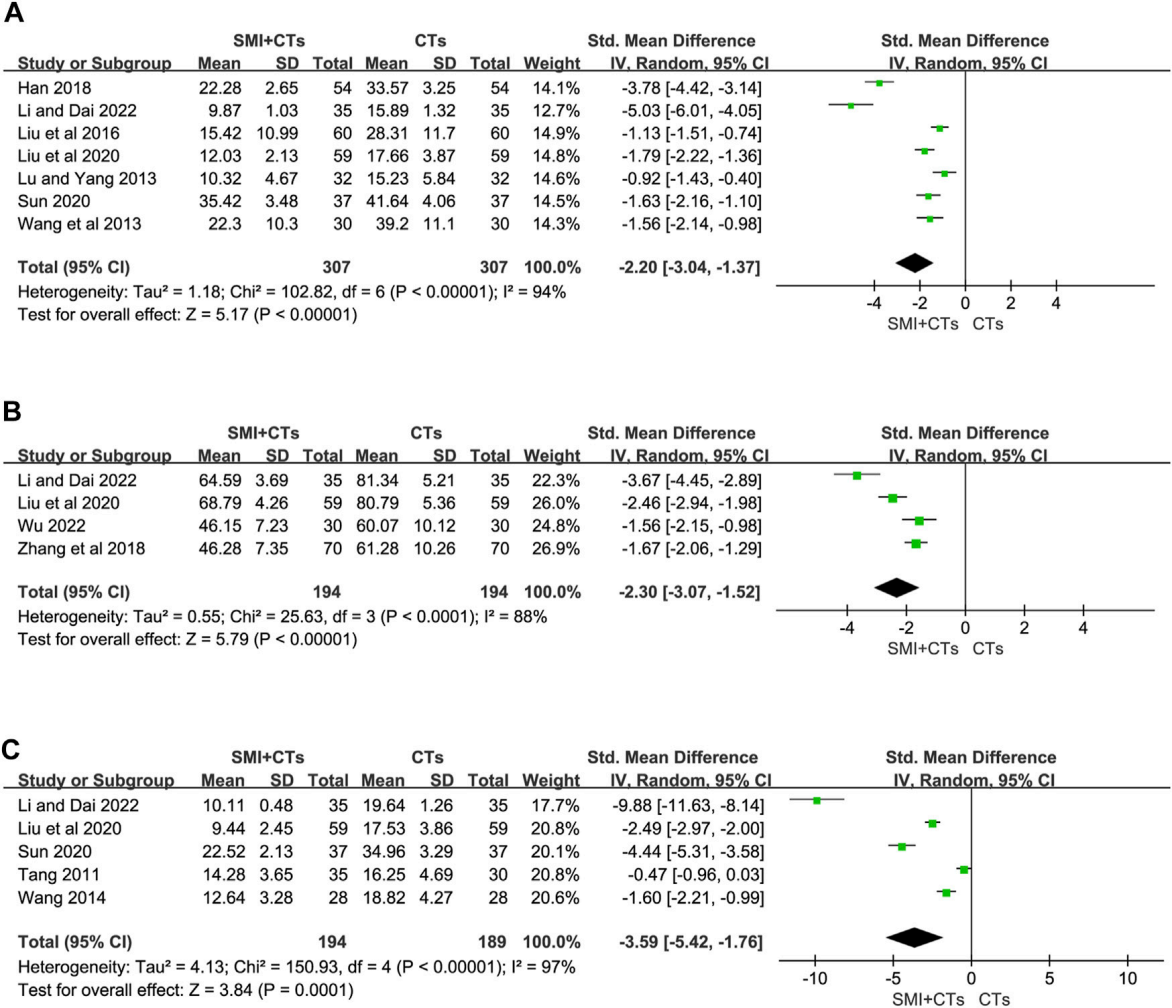
Forest plot for levels of Inflammatory Factors. **(A)** Serum IL-6. **(B)** Serum IL-18. **(C)** Serum hs-CRP.

Serum IL-6 levels were reported in seven studies (Lu and Yang, 2013; Wang et al., 2013; Liu et al., 2016; Han, 2018; Liu et al., 2020; Sun, 2020; Li and Dai, 2022), and serum IL-18 levels were reported in four (Zhang et al., 2018; Liu et al., 2020; Li and Dai, 2022; Wu, 2022), whereas serum hs-CRP levels were reported in five studies (Tang, 2011; Wang, 2014; Liu et al., 2020; Sun, 2020; Li and Dai, 2022). The random-effects model was also adopted because of the significant variability observed across the studies (*p* < 0.0001, I^2^ = 94%; *p* < 0.0001, I^2^ = 88%; *p* < 0.0001, I^2^ = 97%). Due to inconsistencies in the units used across studies, we utilized the standardized mean difference (SMD) as the effect size indicator. The meta-analysis revealed that combining SMI with CTs significantly reduced the serum IL-6 levels (SMD -2.20, 95% CI: 3.04 to −1.37, *p* < 0.00001, Figure 7A), the serum IL-18 levels (SMD -2.30, 95% CI: 3.07 to −1.52, *p* < 0.00001, Figure 7B), and the serum hs-CRP levels (SMD -3.59, 95% CI: 5.42 to −1.76, *p* = 0.0001, Figure 7C) compared to CTs alone. High heterogeneity might stem from variations in the baseline inflammatory index levels among the included cases. As depicted in the forest plot of IL-18, the studies by Li and Dai, and Liu et al. exhibit greater homogeneity, while those by Wu and Zhang et al. also show homogeneity. Egger’s test results indicated no significant publication bias in serum IL-18 and hs-CRP levels (Supplementary Figure S2). However, a potential publication bias was observed in serum IL-6 levels following the Egger’s test (*p* = 0.032) (Supplementary Figure S2). Therefore, we conducted a trim-and-fill calculation, which revealed no need for trimming. Furthermore, A sensitivity analysis was performed to validate the reliability of the meta-analytic findings (Supplementary Figure S1).

##### 3.4.2.5 Levels of hemorheology

**FIGURE 8:**
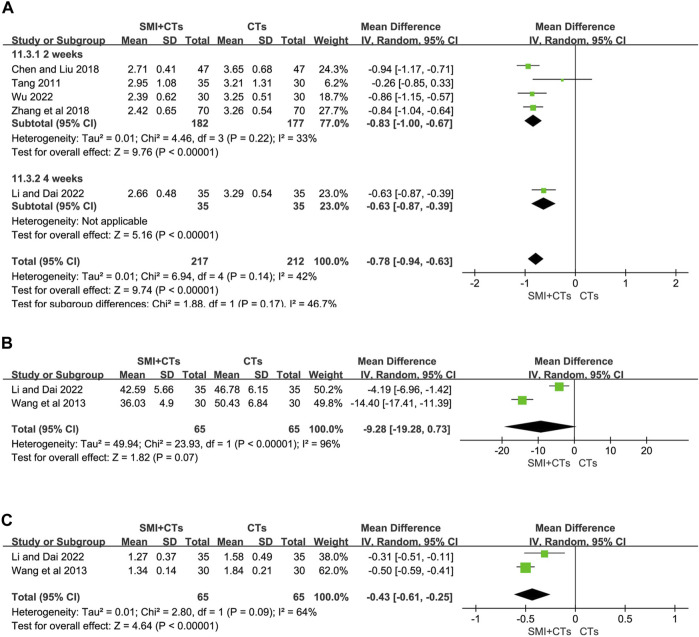
Forest plot for levels of Hemorheology. **(A)** Fibrinogen. **(B)** Hematocrit. **(C)** Plasma viscosity.

Fibrinogen levels were reported in five studies (Tang, 2011; Chen and Liu, 2018; Zhang et al., 2018; Li and Dai, 2022; Wu, 2022), and two (Wang et al., 2013; Li and Dai, 2022) reported hematocrit and plasma viscosity. The random-effects model was also employed in our analysis, with the MD serving as the effect size indicator. We conducted a subgroup analysis on fibrinogen levels depending on the length of the intervention. The fibrinogen levels were significantly lower in groups combining CTs with SMI for 4 weeks (MD -0.83, 95% CI: 1.00 to −0.67, *p* < 0.00001, Figure 8A), as well as for 2 weeks (MD -0.63, 95% CI: 0.87 to −0.39, *p* < 0.00001, Figure 8A) compared to the control group. The findings indicated that the combination of SMI and CTs led to a reduction in plasma viscosity (MD -0.43, 95% CI: 0.61 to −0.25, *p* < 0.00001, Figure 8C). However, the hematocrit decrease did not show a significant difference between patients receiving CTs combined with SMI and those receiving CTs alone (MD -9.28, 95% CI: 19.28 to 0.73, *p* = 0.07, Figure 8C). The results of Egger’s test indicated that there was no substantial publication bias in our study (Supplementary Figure S2). A sensitivity study was performed to confirm the stability and reliability of the meta-analytic results; however, the sources of heterogeneity could not be determined (Supplementary Figure S1). Variations in dose and duration in the trials may have led to this heterogeneity. Further studies are required to enhance the results accuracy and reduce variability.

#### 3.4.3 Adverse events

Adverse events were documented in six out of 17 studies (Hu, 2018; Zhang et al., 2018; Li and Chen, 2019; Liu et al., 2020; Sun, 2020; Wu, 2022). One study (Sun, 2020) found that adverse events were recorded in 1 out of 37 patients in the SMI combining CTs group and 1 out of 37 cases in the CTs alone group. The frequency of adverse events did not change significantly between the two groups (RR = 1.00, 95% CI: 0.06 to 15.40, *p* = 1.00). The precise symptoms were not stated, however.

Five studies documented nine side effects, including elevated transaminase, rash, nausea, bleeding, urticaria, chest tightness, facial blushing, headache, and vomiting. Furthermore, a subgroup analysis based on the symptoms was conducted. As depicted in Table 2, no significant difference was seen in the occurrence of elevated transaminase (RR 0.67, 95% CI: 0.11 to 3.87, *p* = 0.65), rash (RR 0.33, 95% CI: 0.08 to 1.37, *p* = 0.13), nausea (RR 0.57, 95% CI: 0.22 to 1.42, *p* = 0.23), bleeding (RR 0.43, 95% CI: 0.06 to 2.85, *p* = 0.38), urticaria (RR 1.00, 95% CI: 0.18 to 5.69, *p* = 1.00), chest tightness (RR 1.00, 95% CI: 0.23 to 4.35, *p* = 1.00), facial blushing (RR 2.33, 95% CI: 0.35 to 15.58, *p* = 0.38), headache (RR 0.67, 95% CI: 0.12 to 3.85, *p* = 0.65), and vomiting (RR 0.50, 95% CI: 0.05 to 5.37, *p* = 0.57). Additionally, a sensitivity analysis confirmed the stability of the findings obtained from the meta-analysis (Supplementary Figure S1).

**TABLE 2:** Subgroup analysis of the incidence of adverse events.

Types of adverse events	No.	RR	95% CI	I^2^ (%)	*p*-value for heterogeneity test	*p*-value for overall effect
Elevated transaminase	2	0.67	0.11 to 3.87	0	0.71	0.65
Rash	3	0.33	0.08 to 1.37	0	0.76	0.13
Nausea	3	0.57	0.22 to 1.42	0	0.72	0.23
Bleeding	2	0.43	0.06 to 2.85	0	0.84	0.38
Urticaria	2	1.00	0.18 to 5.69	0	0.38	1.00
Chest tightness	3	1.00	0.23 to 4.35	0	0.68	1.00
Facial Blushing	2	2.33	0.35 to 15.58	0	0.43	0.38
Headache	1	0.67	0.12 to 3.85	-	-	0.65
Vomiting	1	0.50	0.05 to 5.37	-	-	0.57

### 3.5 Publication bias

**FIGURE 9:**
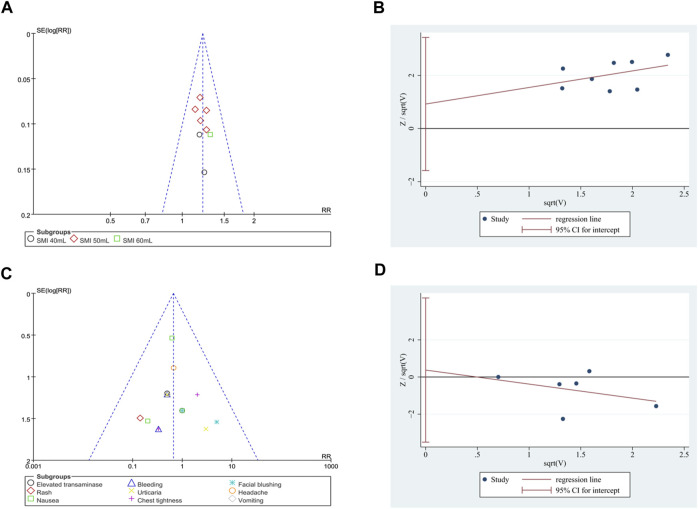
Publication bias. **(A)** Funnel plot of total effective rate. **(B)** The Harbord’s test on total effective rate. **(C)** Funnel plot of the incidence of adverse events. **(D)** The Harbord’s test on the incidence of adverse events.

Due to the low heterogeneity and limited number of studies available for analysis, the Harbord test was adopted. The results indicated no obvious publication bias for total effective rate (*p* = 0.403; Figure 9B) and the incidence of adverse events (*p* = 0.802; Figure 9D). Additionally, the funnel plot of the total effective rate and the incidence of adverse events did not show visual asymmetry (Figures 9A, C).

### 3.6 Quality of evidence

The GRADE system was utilized to methodically assess the quality of 10 outcome indicators (Table 3). The findings indicated moderate-quality evidence for total effective rate, BI, and fibrinogen levels. Additionally, low-quality evidence was identified for NIHSS score and serum IL-6 levels, followed by very low evidence for mRS, serum IL-18 levels, serum hs-CRP levels, hematocrit, and plasma viscosity. The significant risks of deviations and reported deviations are attributed to the following five factors: 1) a potential for publication bias was present; 2) the heterogeneity among the studies is significant (*p* < 0.1, I^2^ > 50%); 3) the 95% confidence interval includes 1; 4) sample sizes of <400 were included; and 5) the number of included RCTs is fewer than 5. More extensive samples and well-conducted RCTs are needed to improve the evidence on the effectiveness of SMI in treating AIS owing to limitations in the present study.

**TABLE 3:** GRADE summary of evidence.

No	Design	Certainty assessment					Results summary				Importance
		Risk of bias	Inconsistency	Indirectness	Imprecision	Other considerations	No. of patients		Effect (95% CI)			Quality
							T	C	Relative	Absolute		
Total effective rate												
8	RCT	Serious^a^	Not serious	Not serious	Not serious	Not serious	366	366	RR 1.22 (1.14–1.3)	-	⊕⊕⊕Ο Moderate	Critical
NIHSS score												
13	RCT	Serious^a^	Serious^b^	Not serious	Not serious	Not serious	638	638	-	MD 3.05 lower (3.85–2.24 lower)	⊕⊕ΟΟ Low	Critical
Barthel index (BI)												
5	RCT	Serious^a^	Not serious	Not serious	Not serious	Not serious	235	235	-	MD 12.18 higher (10.3–14.06 higher)	⊕⊕⊕Ο Moderate	Critical
Modified Rankin Scale (mRS)												
3	RCT	Serious^a^	Serious^b^	Not serious	Serious^d^	Serious^e^	160	160	-	MD 0.68 lower (0.86–0.49 lower)	⊕ΟΟΟ Very low	Critical
Serum IL-6 levels												
7	RCT	Serious^a^	Serious^b^	Not serious	Not serious	Not serious	307	307	-	SMD 2.2 lower (3.04–1.37 lower)	⊕⊕ΟΟ Low	Important
Serum IL-18 levels												
4	RCT	Serious^a^	Serious^b^	Not serious	Serious^d^	Serious^e^	194	194	-	SMD 2.3 lower (3.07–1.52 lower)	⊕ΟΟΟ Very low	Important
Serum hs-CRP levels												
5	RCT	Serious^a^	Serious^b^	Not serious	Serious^d^	Not serious	194	189	-	SMD 3.59 lower (5.42–1.76 lower)	⊕ΟΟΟ Very low	Important
Fibrinogen levels												
5	RCT	Serious^a^	Not serious	Not serious	Not serious	Not serious	217	212	-	MD 0.78 lower (0.94–0.63 lower)	⊕⊕⊕Ο Moderate	Important
Hematocrit												
2	RCT	Serious^a^	Serious^b^	Not serious	Serious^c,d^	Serious^e^	65	65	-	MD 9.28 lower (19.28–0.73 lower)	⊕ΟΟΟ Very low	Important
Plasma viscosity												
2	RCT	Serious^a^	Serious^b^	Not serious	Serious^d^	Serious^e^	65	65	-	MD 0.43 lower (0.61–0.25 lower)	⊕ΟΟΟ Very low	Important

Risk of bias.

^a^ A potential for publication bias was present.

Inconsistency.

^b^ The heterogeneity among studies is significant (*p* < 0.1, I2 > 50%).

Imprecision.

^c^ The 95% confidence interval includes 1.

^d^ Sample sizes of less than 400 were included.

Other.

^e^ The number of included RCTs, is fewer than 5.

## 4 Discussion

### 4.1 Summary of findings

This systematic review discusses the efficacy and safety of SMI in treating AIS. A total of 17 RCTs were considered, with 1,603 participants. The treatment with SMI appears effective and safe, as it can lower levels of mRS, NIHSS scores, and inflammatory markers, while improving overall effective rate, BI, and cerebral hemodynamics without increasing the occurrence of adverse reactions.

Our subgroup analysis revealed that, compared to CTs, the combination of 20, 40, 50, and 60 mL SMI with CTs significantly enhanced overall effective rate and BI, while reducing NIHSS score. This indicates that within an appropriate dosage range, the combination of SMI effectively alleviates clinical symptoms, facilitates neurological function recovery, and enhances daily living abilities in patients. Moreover, our study highlights the necessity for future investigations to explore the optimal dosage regimens for SMI in AIS treatment. Through systematic evaluation of the effectiveness and safety of various dosage regimens, we can deepen our understanding of the therapeutic potential of SMI and optimize its clinical application. Regarding the intervention duration, SMI combined with CTs treatment for 2 and 4 weeks significantly decreased fibrinogen levels. Additionally, these findings suggest that the effect of SMI is sustained and rapid. The meta-analysis found that SMI did not increase the incidence of patients experiencing increased transaminases, rash, nausea, chest tightness, bleeding, urticaria, facial flushing, headache, or vomiting in patients. However, this implies the necessity of closely monitoring the coagulation function, transaminase levels, and allergic reactions in patients receiving SMI.

Sensitivity analysis suggests that the combined results are robust. However, the origin of heterogeneity cannot be determined. The restricted number of included studies, small sample size, and large variability in early laboratory markers across patients may all contribute to significant heterogeneity. Nonetheless, due to the absence of available data, further discussion was not pursued in the article.

### 4.2 Interpretation of findings

Inflammation is involved in the occurrence, progression, and development of AIS, which can induce thrombosis. This plays a crucial role in the pathophysiology of ischemic stroke and is a primary focus for the creation of novel stroke treatments (Jiang et al., 2021; Zuo et al., 2021). Increased levels of the pro-inflammatory factor IL-6 induced by ischemia often cause endothelial cells to express adhesion factors and chemokines, which in turn activate monocytes to release relevant inflammatory cytokines, leading to the formation of atherosclerotic thrombosis (Lei et al., 2014). Prospective studies have shown that high levels of IL-6 in community-dwelling individuals are directly linked to an increased risk of developing ischemic stroke in the long run, regardless of traditional vascular risk factors (Papadopoulos et al., 2022). Moreover, higher CRP levels have been demonstrated to independently predict survival after ischemic stroke and functional outcomes following thrombolytic stroke (Nishi et al., 2020). According to the results of this meta-analysis, combination therapy with SMI and CTs may lower blood levels of IL-6, IL-18, and hs-CRP compared to CTs alone. Thrombosis typically develops from coagulation disorders induced by vascular endothelial cell injury, platelet aggregation, and alterations in hemorheology (Ge et al., 2018). Fibrinogen, a vascular inflammatory mediator, has recently been identified as a driver of the neurodegenerative process in cerebrovascular stroke (Wang et al., 2020). The meta-analysis showed that combining SMI with CTs effectively reduces fibrinogen levels and plasma viscosity, leading to an improvement in hemorheological parameters. We refer to the ConPhyMP statement to ensure the rigor of our conclusions (Heinrich et al., 2022). The main botanical drugs of SMI are *P. ginseng* C.A. Mey. [Araliaceae; Radix ginseng rubra] and *O. japonicus* (Thunb.) Ker Gawl. [Asparagaceae; Ophiopogonis Radix], which are utilized to invigorate Qi and nourish Yin. Treatment with *Ginsenoside Rg1* inhibits the interaction between CKLF1 and CCR5 after ischemic stroke, reducing pyroptosis generated by the CKLF1/CCR5 axis (Long et al., 2024). Ginsenosides also inhibit thrombogenesis by interfering with multiple signaling pathways involved in platelet aggregation, thereby inhibiting the production of thromboxane A2 (TXA2) and phosphorylation of MAPKs (Zhou et al., 2019; Luo et al., 2020) found that different dosage groups of *O. japonicus polysaccharides* can inhibit NF-κB activation in the B signaling pathway, thereby reducing the oxidative stress response, alleviating macrophage damage, and decreasing the secretion of inflammatory factors. In summary, SMI can exert neuroprotective effects on patients with AIS by reducing the levels of inflammatory factors and improving cerebral hemodynamics.

### 4.3 Advantages and limitations

A study conducted in 2017 showed that SMI is effective and safe for the treatment of AIS. However, compared with previous studies, ours possesses the following advantages. Firstly, integrating efficacy evaluation criteria based on the decline rate of NIHSS score in our study enables a more comprehensive assessment of the effectiveness of SMI. This not only reduces inconsistency but also enhances the credibility of the results. Moreover, by excluding interventions from other traditional Chinese medicine therapies, we minimized the impact on the accuracy of results, thus providing stronger confirmation of the efficacy of SMI. Furthermore, we employed a subgroup analysis to investigate the effects of medication dosage and duration on efficacy, and further explored the sources of heterogeneity. Finally, we utilized ROB 2.0 and the GRADE profiler to evaluate the risk of bias and the quality of evidence, enhancing the credibility of the findings.

Nevertheless, our study has some limitations. First, most of the included studies showed a risk of bias, reducing the trustworthiness of the findings. Second, although we conducted a sensitivity analysis to assess the stability of the results, caution is warranted in interpreting these findings due to factors such as small sample size, high heterogeneity, and potential publication bias. Third, the study population included in this analysis exclusively consisted of individuals from China, neglecting the potential influence of race and population diversity. This limitation may hinder the broader promotion and application of SMI. Fourth, IVT and EVTs are crucial treatment options for acute cerebral infarction. However, due to their potential impact on outcome consistency, we chose not to include studies in this area. Fifth, the scientific rigor of the efficacy evaluation criteria we have selected may still be subject to debate, particularly considering the variations in stroke severity among participants. Finally, the studies included in our analysis lacked allocation concealment or blinding, and some failed to adequately describe methods for generating random sequences. Consequently, the overall methodological quality was deemed to be low.

### 4.4 Implications for future studies

We demonstrate the effectiveness of SMI for AIS; however, this has certain limitations that can inspire future research designs. Firstly, considering the extended recovery period of AIS, future clinical studies should incorporate a longer follow-up duration to confirm the potential benefits of SMI in enhancing AIS prognosis. Secondly, there is currently limited research on the combination of SMI with IVT and EVT, necessitating further investigation in this area in the future. Thirdly, when possible, blinding should be implemented using random and covert allocation techniques, and suitable methods to determine sample size should be used. Fourthly, the test report should adhere to the Uniform Standards of Reporting Trials (CONSORT) statement, facilitating the acquisition of more comprehensive information. Finally, the selection of outcome indicators should adhere to uniform standards to comprehensively evaluate the effectiveness and safety of SMI.

## 5 Conclusion

Overall, this meta-analysis suggests that the combination of SMI with CTs appears to be more effective in treating AIS. Furthermore, none of the adverse effects were severe enough to be considered life-threatening. However, larger, higher-quality trials are required to further corroborate and validate the clinical efficacy and safety of SMI in the treatment of AIS, given the substantial heterogeneity and low quality of evidence.

## Data Availability

The datasets presented in this study can be found in online repositories. The names of the repository/repositories and accession number(s) can be found in the article/Supplementary Material.

## Abbreviations

AIS, Acute ischemic stroke; BI, Barthel index; CI, Confidence intervals; CTs, Conventional treatments; IVT, Intravenous thrombolysis; MD, Mean difference; NIHSS, National Institute of Health Stroke Scale Score; RCTs, Randomized controlled trials; SMD, Standardized mean difference; SMI, Shenmai injection.
