# Supplementary material for: Efficacy and safety of Shenmai injection for acute ischemic stroke: a systematic review and meta-analysis
Source: Front Pharmacol. 2024 Jun 4;15:1394936. doi: 10.3389/fphar.2024.1394936 (PMC11184089; doi:10.3389/fphar.2024.1394936)
Supplement: Supplementary file 1 [file DataSheet1.docx]

Supplementary Material

[Supplementary Table S1 Search strategy 2](#_Toc161239097)

[Supplementary Table S2 The details of SMI in the included study. 6](#_Toc161239098)

[Supplementary Table S3 The principal chemical constituents of SMI under fingerprint methods. 9](#_Toc161239099)

[Supplementary Figure S1 Sensitivity analysis 10](#_Toc161239100)

[Supplementary Figure S2 Egger's test 13](#_Toc161239101)

[Supplementary Table S4 The PRISMA checklist of this meta-analysis 16](#_Toc161239102)

#

# Supplementary Table S1 Search strategy

| **Databases** | **Search items** | **Number** |
| --- | --- | --- |
| **PubMed** | # l:"Shenmai injection"[Title/Abstract] OR "Shenmai"[Title/Abstract]  #2:"Ischemic Stroke"[Mesh] OR "Ischemic Strokes"[Title/Abstract] OR "Stroke, Ischemic" [Title/Abstract] OR "Ischaemic Stroke" [Title/Abstract] OR "Ischaemic Strokes" [Title/Abstract] OR "Stroke, Ischaemic" [Title/Abstract] OR "Cryptogenic Ischemic Stroke" [Title/Abstract] OR "Cryptogenic Ischemic Strokes"[Title/Abstract] OR "Ischemic Stroke, Cryptogenic"[Title/Abstract] OR "Stroke, Cryptogenic Ischemic"[Title/Abstract] OR "Cryptogenic Stroke"[Title/Abstract] OR "Cryptogenic Strokes"[Title/Abstract] OR "Stroke, Cryptogenic"[Title/Abstract] OR "Cryptogenic Embolism Stroke"[Title/Abstract] OR "Cryptogenic Embolism Strokes"[Title/Abstract] OR "Embolism Stroke, Cryptogenic"[Title/Abstract] OR "Stroke, Cryptogenic Embolism"[Title/Abstract] OR "Wake-up Stroke"[Title/Abstract] OR "Stroke, Wake-up"[Title/Abstract] OR "Wake up Stroke"[Title/Abstract] OR "Wake-up Strokes"[Title/Abstract] OR "Acute Ischemic Stroke"[Title/Abstract] OR "Acute Ischemic Strokes"[Title/Abstract] OR "Ischemic Stroke, Acute"[Title/Abstract] OR “Stroke, Acute Ischemic"[Title/Abstract]  #3:"randomized controlled trial"[Title/Abstract] OR "randomized"[Title/Abstract] OR "placebo"[Title/Abstract]  #4:#1 AND #2 AND #3 | 1 |
| **Cochrane Library** | #1MeSH descriptor: [Ischemic Stroke] explode all trees  #2(Ischemic Strokes):ab,kw,ti OR (Stroke, Ischemic):ab,kw,ti OR (Ischaemic Stroke):ab,kw,ti OR (Ischaemic Strokes):ab,kw,ti OR (Stroke, Ischaemic):ab,kw,ti OR (Cryptogenic Ischemic Stroke):ab,kw,ti OR (Cryptogenic Ischemic Strokes):ab,kw,ti OR (Ischemic Stroke, Cryptogenic):ab,kw,ti OR (Stroke, Cryptogenic Ischemic):ab,kw,ti OR (Cryptogenic Stroke):ab,kw,ti OR (Cryptogenic Strokes):ab,kw,ti OR (Stroke, Cryptogenic):ab,kw,ti OR (Cryptogenic Embolism Stroke):ab,kw,ti OR (Cryptogenic Embolism Strokes):ab,kw,ti OR (Embolism Stroke, Cryptogenic):ab,kw,ti OR (Stroke, Cryptogenic Embolism):ab,kw,ti OR (Wake-up Stroke):ab,kw,ti OR (Stroke, Wake-up):ab,kw,ti OR (Wake up Stroke):ab,kw,ti OR (Wake-up Strokes):ab,kw,ti OR (Acute Ischemic Stroke):ab,kw,ti OR (Acute Ischemic Strokes):ab,kw,ti OR (Ischemic Stroke, Acute):ab,kw,ti OR (Stroke, Acute Ischemic):ab,kw,ti  #3(shenmai injection):ab,kw,ti OR (shenmai):ab,kw,ti  #4(randomized controlled trial):ab,kw,ti OR (randomized):ab,kw,ti OR (placebo):ab,kw,ti  #5:#1 AND #2 AND #3 AND #4 | 0 |
| **Web of science** | #1TS=(Shenmai injection OR Shenmai )  #2TS=(acute ischemic stroke OR ischemic strokes OR stroke, ischemic OR ischaemic stroke OR ischaemic strokes OR stroke, ischaemic OR cryptogenic ischemic stroke OR cryptogenic ischemic strokes OR ischemic stroke, cryptogenic OR stroke, cryptogenic ischemic OR cryptogenic stroke OR cryptogenic strokes OR stroke, cryptogenic OR cryptogenic embolism stroke OR cryptogenic embolism strokes OR embolism stroke, cryptogenic OR stroke, cryptogenic embolism OR wake-up stroke OR stroke, wake-up OR wake up stroke OR wake-up strokes OR acute ischemic stroke OR acute ischemic strokes OR ischemic stroke, acute OR stroke, acute ischemic)  #3TS=(randomized controlled trial OR randomized OR placebo)  #4:#1 AND #2 AND #3 | 2 |
| **Embase** | #1 'acute ischemic stroke'/exp OR 'acute ischemic stroke'  #2 'shenmai injection':ab,kw,ti OR 'shenmai':ab,kw,ti  #3 'ischemic strokes':ab,kw,ti OR 'stroke, ischemic':ab,kw,ti OR 'ischaemic stroke':ab,kw,ti OR 'ischaemic strokes':ab,kw,ti OR 'stroke, ischaemic':ab,kw,ti OR 'cryptogenic ischemic stroke':ab,kw,ti OR 'cryptogenic ischemic strokes':ab,kw,ti OR 'ischemic stroke, cryptogenic':ab,kw,ti OR 'stroke, cryptogenic ischemic':ab,kw,ti OR 'cryptogenic stroke':ab,kw,ti OR 'cryptogenic strokes':ab,kw,ti OR 'stroke, cryptogenic':ab,kw,ti OR 'cryptogenic embolism stroke':ab,kw,ti OR 'cryptogenic embolism strokes':ab,kw,ti OR 'embolism stroke, cryptogenic':ab,kw,ti OR 'stroke, cryptogenic embolism':ab,kw,ti OR 'wake-up stroke':ab,kw,ti OR 'stroke, wake-up':ab,kw,ti OR 'wake up stroke':ab,kw,ti OR 'wake-up strokes':ab,kw,ti OR 'acute ischemic stroke':ab,kw,ti OR 'acute ischemic strokes':ab,kw,ti OR 'ischemic stroke, acute':ab,kw,ti OR 'stroke, acute ischemic':ab,kw,ti  #4 'randomized controlled trial ':ab,kw,ti OR 'randomized':ab,kw,ti OR 'placebo':ab,kw,ti  #5:#1 AND #2 AND #3 AND #4 | 1 |
| **CNKI** | (SU='急性脑梗死'+'急性缺血性卒中'+'脑梗死'+'卒中') AND (SU='参麦注射液'+'参麦') | 76 |
| **Wanfang** | （题名或关键词=参麦注射液 OR 参麦) AND （题名或关键词=急性脑梗死 OR 急性缺血性卒中 OR 缺血性卒中 OR 脑梗死） | 122 |
| **VIP** | （题名或关键词=参麦注射液 OR 参麦) AND （题名或关键词=急性脑梗死 OR 急性缺血性卒中 OR 缺血性卒中 OR 脑梗死） | 138 |
| **Sinomed** | ( "参麦注射液"[常用字段:智能] OR "参麦"[常用字段:智能]) AND( "急性脑梗死"[常用字段:智能] OR "急性缺血性卒中"[常用字段:智能] OR "缺血性卒中"[常用字段:智能] OR "脑梗死"[常用字段:智能]) | 148 |

# Supplementary Table S2 The details of SMI in the included study.

| **Study** | **Name** | **Source** | **Composition** | **Quality control reported?**  **(Y/N)** | **Chemical analysis reported?**  **(Y/N)** | **Actions** |
| --- | --- | --- | --- | --- | --- | --- |
| Li and Dai.  (2022) | Shenmai  injection | Hebei Shenwei Pharmaceutical Co., Ltd | - Panax ginseng C.A.Mey. [Araliaceae; Radix ginseng rubra],100g - Ophiopogon japonicus (Thunb.) Ker Gawl. [Asparagaceae; Ophiopogonis radix], 100g | Y – Prepared according to National Drug Standards of China Food and Drug Administration (WS3-B-3428-98-2010Z) | N | Tonifying qi and preventing exhaustion, nourishing yin and promoting fluid |
| Sun.  (2020) | Shenmai  injection | Enbipu Pharmaceutical Co., Ltd. of Shiyao Group | - Panax ginseng C.A.Mey. [Araliaceae; Radix ginseng rubra],100g - Ophiopogon japonicus (Thunb.) Ker Gawl. [Asparagaceae; Ophiopogonis radix], 100g | Y – Prepared according to National Drug Standards of China Food and Drug Administration (WS3-B-3428-98-2010Z) | N |  |
| Liu et al.  (2020) | Shenmai  injection | N | - Panax ginseng C.A.Mey. [Araliaceae; Radix ginseng rubra],100g - Ophiopogon japonicus (Thunb.) Ker Gawl. [Asparagaceae; Ophiopogonis radix], 100g | Y – Prepared according to National Drug Standards of China Food and Drug Administration (WS3-B-3428-98-2010Z) | N |  |
| Li and Chen.（2019） | Shenmai  injection | Sichuan Shenghe Pharmaceutical Co., Ltd | - Panax ginseng C.A.Mey. [Araliaceae; Radix ginseng rubra],100g - Ophiopogon japonicus (Thunb.) Ker Gawl. [Asparagaceae; Ophiopogonis radix], 100g | Y – Prepared according to National Drug Standards of China Food and Drug Administration (WS3-B-3428-98-2010Z) | N |  |
| Chen.  （2019） | Shenmai  injection | Hebei Shenwei Pharmaceutical Co., Ltd | - Panax ginseng C.A.Mey. [Araliaceae; Radix ginseng rubra],100g - Ophiopogon japonicus (Thunb.) Ker Gawl. [Asparagaceae; Ophiopogonis radix], 100g | Y – Prepared according to National Drug Standards of China Food and Drug Administration (WS3-B-3428-98-2010Z) | N |  |
| Zhang et al.  （2018） | Shenmai  injection | Dali Pharmaceutical  Co., Ltd | - Panax ginseng C.A.Mey. [Araliaceae; Radix ginseng rubra],100g - Ophiopogon japonicus (Thunb.) Ker Gawl. [Asparagaceae; Ophiopogonis radix], 100g | Y – Prepared according to National Drug Standards of China Food and Drug Administration (WS3-B-3428-98-2010Z) | N |  |
| Wu et al.  （2018） | Shenmai  injection | Zhengda Qingchun Bao Pharmaceutical Co., Ltd | - Panax ginseng C.A.Mey. [Araliaceae; Radix ginseng rubra],100g - Ophiopogon japonicus (Thunb.) Ker Gawl. [Asparagaceae; Ophiopogonis radix], 100g | Y – Prepared according to National Drug Standards of China Food and Drug Administration (WS3-B-3428-98-2010Z) | N |  |
| Liang et al.  （2018） | Shenmai  injection | Zhengda Qingchun Bao Pharmaceutical Co., Ltd | - Panax ginseng C.A.Mey. [Araliaceae; Radix ginseng rubra],100g - Ophiopogon japonicus (Thunb.) Ker Gawl. [Asparagaceae; Ophiopogonis radix], 100g | Y – Prepared according to National Drug Standards of China Food and Drug Administration (WS3-B-3428-98-2010Z) | N |  |
| Wu.  （2022） | Shenmai  injection | N | - Panax ginseng C.A.Mey. [Araliaceae; Radix ginseng rubra],100g - Ophiopogon japonicus (Thunb.) Ker Gawl. [Asparagaceae; Ophiopogonis radix], 100g | Y – Prepared according to National Drug Standards of China Food and Drug Administration (WS3-B-3428-98-2010Z) | N |  |
| Hu.  （2018） | Shenmai  injection | Sichuan Chuanda Huaxi Pharmaceutical Co., Ltd | - Panax ginseng C.A.Mey. [Araliaceae; Radix ginseng rubra],100g - Ophiopogon japonicus (Thunb.) Ker Gawl. [Asparagaceae; Ophiopogonis radix], 100g | Y – Prepared according to National Drug Standards of China Food and Drug Administration (WS3-B-3428-98-2010Z) | N |  |
| Han.  （2018） | Shenmai  injection | Zhengda Qingchun Bao Pharmaceutical Co., Ltd | - Panax ginseng C.A.Mey. [Araliaceae; Radix ginseng rubra],100g - Ophiopogon japonicus (Thunb.) Ker Gawl. [Asparagaceae; Ophiopogonis radix], 100g | Y – Prepared according to National Drug Standards of China Food and Drug Administration (WS3-B-3428-98-2010Z) | N |  |
| Chen and Liu.  （2018） | Shenmai  injection | Zhengda Qingchun Bao Pharmaceutical Co., Ltd | - Panax ginseng C.A.Mey. [Araliaceae; Radix ginseng rubra],100g - Ophiopogon japonicus (Thunb.) Ker Gawl. [Asparagaceae; Ophiopogonis radix], 100g | Y – Prepared according to National Drug Standards of China Food and Drug Administration (WS3-B-3428-98-2010Z) | N |  |
| Liu et al.  (2016) | Shenmai  injection | N | - Panax ginseng C.A.Mey. [Araliaceae; Radix ginseng rubra],100g - Ophiopogon japonicus (Thunb.) Ker Gawl. [Asparagaceae; Ophiopogonis radix], 100g | Y – Prepared according to National Drug Standards of China Food and Drug Administration (WS3-B-3428-98-2010Z) | N |  |
| Wang.  （2014） | Shenmai  injection | N | - Panax ginseng C.A.Mey. [Araliaceae; Radix ginseng rubra],100g - Ophiopogon japonicus (Thunb.) Ker Gawl. [Asparagaceae; Ophiopogonis radix], 100g | Y – Prepared according to National Drug Standards of China Food and Drug Administration (WS3-B-3428-98-2010Z) | N |  |
| Wang et al.  (2013) | Shenmai  injection | Zhengda Qingchun Bao Pharmaceutical Co., Ltd | - Panax ginseng C.A.Mey. [Araliaceae; Radix ginseng rubra],100g - Ophiopogon japonicus (Thunb.) Ker Gawl. [Asparagaceae; Ophiopogonis radix], 100g | Y – Prepared according to National Drug Standards of China Food and Drug Administration (WS3-B-3428-98-2010Z) | N |  |
| Lu and Yang.  （2013） | Shenmai  injection | Hebei Shenwei Pharmaceutical Co., Ltd | - Panax ginseng C.A.Mey. [Araliaceae; Radix ginseng rubra],100g - Ophiopogon japonicus (Thunb.) Ker Gawl. [Asparagaceae; Ophiopogonis radix], 100g | Y – Prepared according to National Drug Standards of China Food and Drug Administration (WS3-B-3428-98-2010Z) | N |  |
| Tang.  （2011） | Shenmai  injection | N | - Panax ginseng C.A.Mey. [Araliaceae; Radix ginseng rubra],100g - Ophiopogon japonicus (Thunb.) Ker Gawl. [Asparagaceae; Ophiopogonis radix], 100g | Y – Prepared according to National Drug Standards of China Food and Drug Administration (WS3-B-3428-98-2010Z) | N |  |

# Supplementary Table S3 The principal chemical constituents of SMI under fingerprint methods.

| **Literature sources** | **fingerprinting methods** | **chemical constituents** |
| --- | --- | --- |
| (Wu et al., 2014) | HPLC-MS/MS | 1.Radix ginseng rubra  Ginsenoside Rg1, ginsenoside Re, ginsenoside Rb1  2. Ophiopogonis radix  maitake saponin D, maitake saponin D′, methylmaitake dihydroflavone A and methylmaitake dihydroflavone B |
| (Li et al., 2022) | ^1^H-NMR | 1.Amino acids (isoleucine, valine, alanine, glutamic acid, glutamine, pyroglutamic acid, proline)  2. Small molecule organic acids (pyruvic acid, malic acid, succinic acid, malonic acid, γ- Aminobutyric acid, lactic acid, acetic acid, formic acid)  3. alkaloid (choline)  4. Carbohydrates (glucose, fructose, sucrose, maltose)  5. Nucleotides (Adenosine, uridine)  6. saponins (ginsenosides Rb1, Rb2, Rc, Rd, Re, Rf, Rg1)  7. Other ingredients (polysorbate 80, ethanol, acetone) |

References

Wu, Y., Wei, X., Zhang, L., Yu, Z., Ren, B., Qi, J., and Dong, Z. (2014) Simultaneous determination of seven components in Shenmai Injection by HPLC-MS/MS. *Chin. Tradit. Herbal. Drugs.* 45(18):2625-2630. doi: 10.7501/j.issn.0253-2670.2014.18.010

Li,W., Yang, J., Zhao, F., Pan, J., and Qu, H. (2022). Fingerprint of Shenmai Injection based on 1H-NMR technique. Chin J. Chin. Mater. Med. 47(03):581-586. doi:10.19540/j.cnki.cjcmm.20211108.303.

# Supplementary Figure S1 Sensitivity analysis

1.Sensitivity analysis for Total effective rate(A), NIHSS score(B), BI(C) and mRS(D).


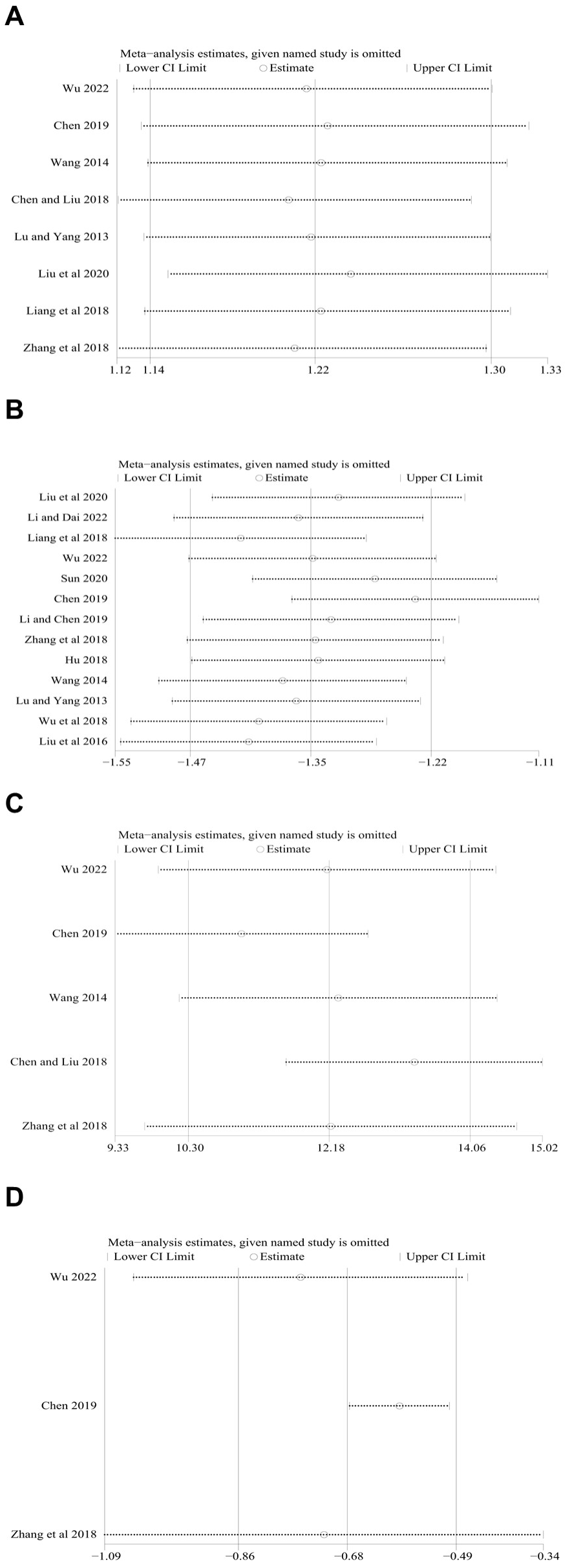


2.Sensitivity analysis for Serum IL-6(A), Serum IL-18(B), Serum hs-CRP(C) and Fibrinogen levels(D).


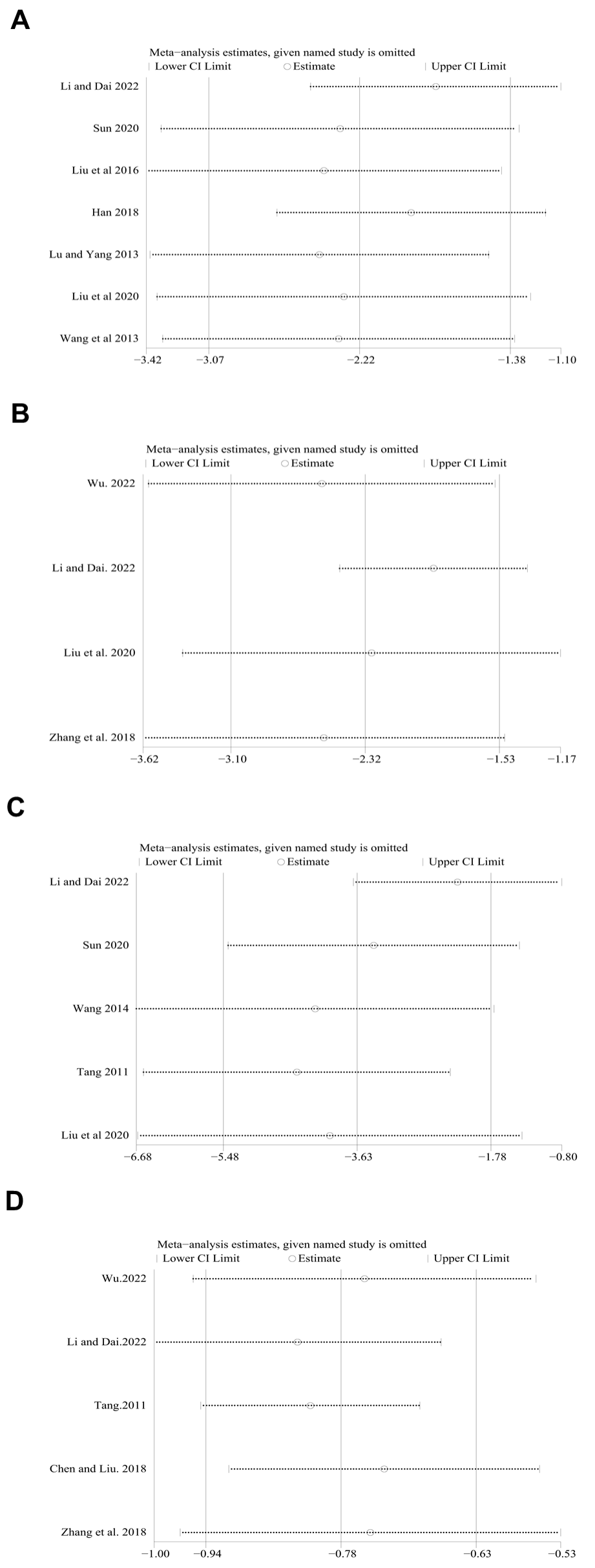


3.Sensitivity analysis for Hematocrit(A), Plasma viscosity(B) and Adverse events(C).


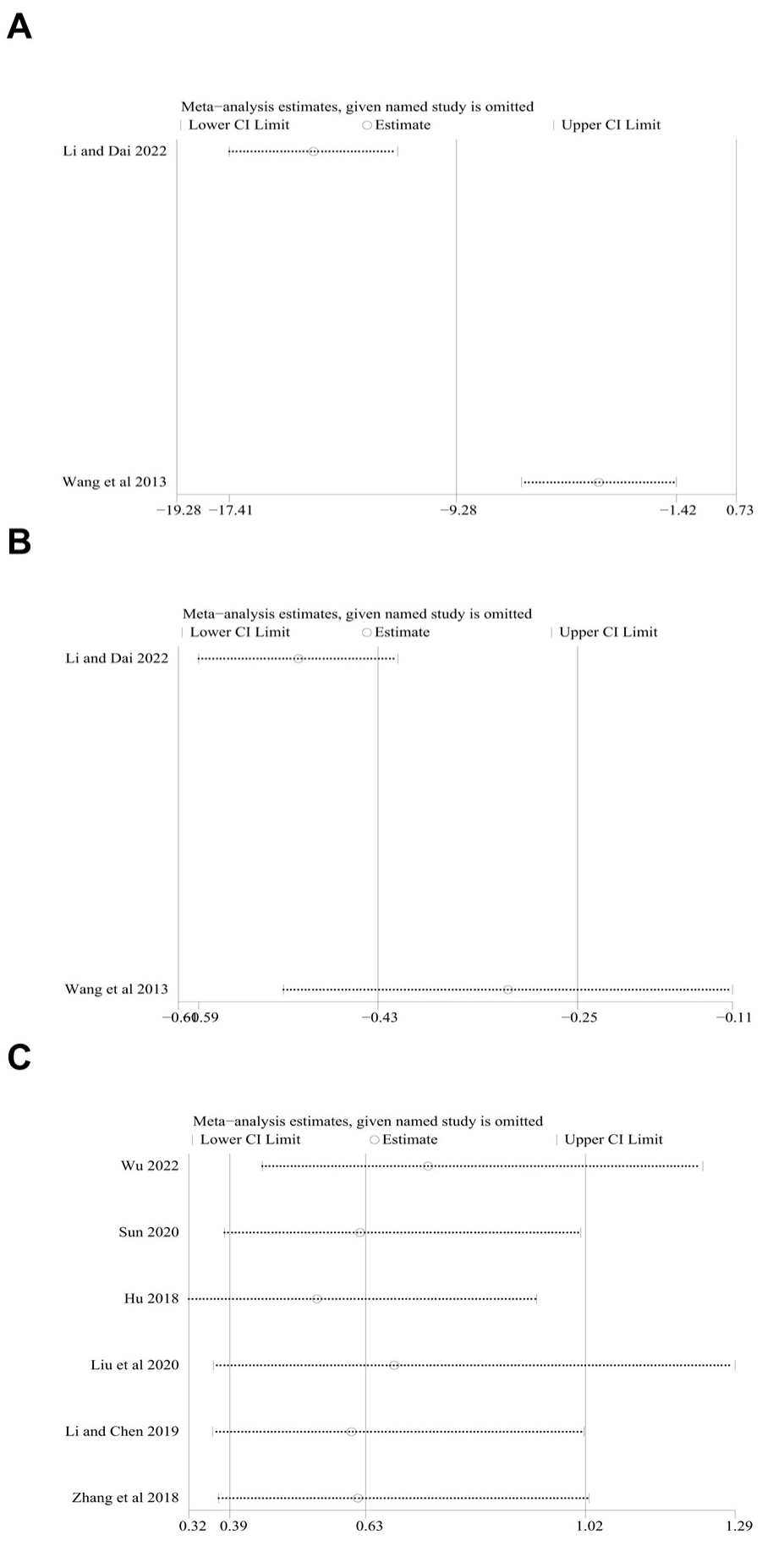


# Supplementary Figure S2 Egger's test

1. Egger's test for NIHSS score(A), BI(B) and mRS(C).


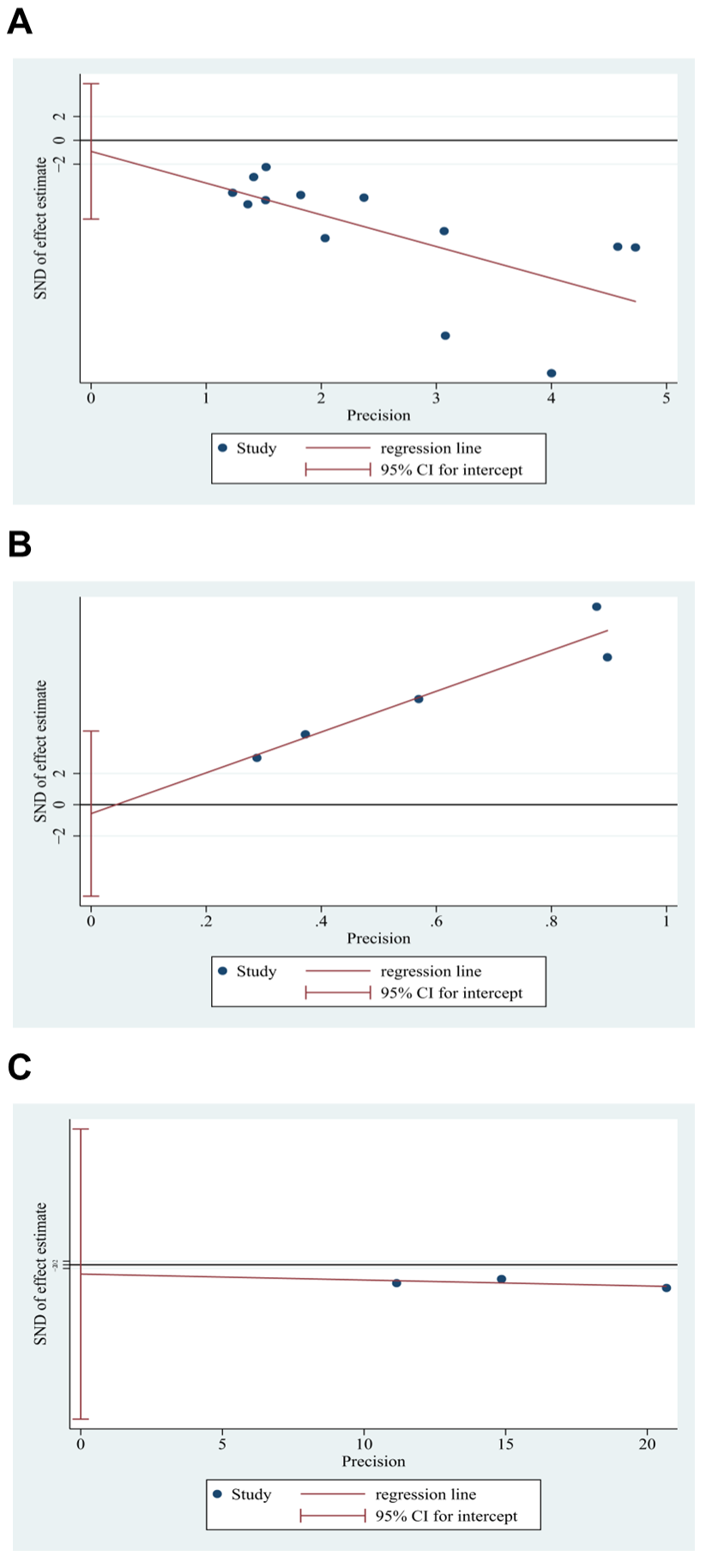


2. Egger's test for Serum IL-6(A), Serum IL-18(B) and Serum hs-CRP(C).


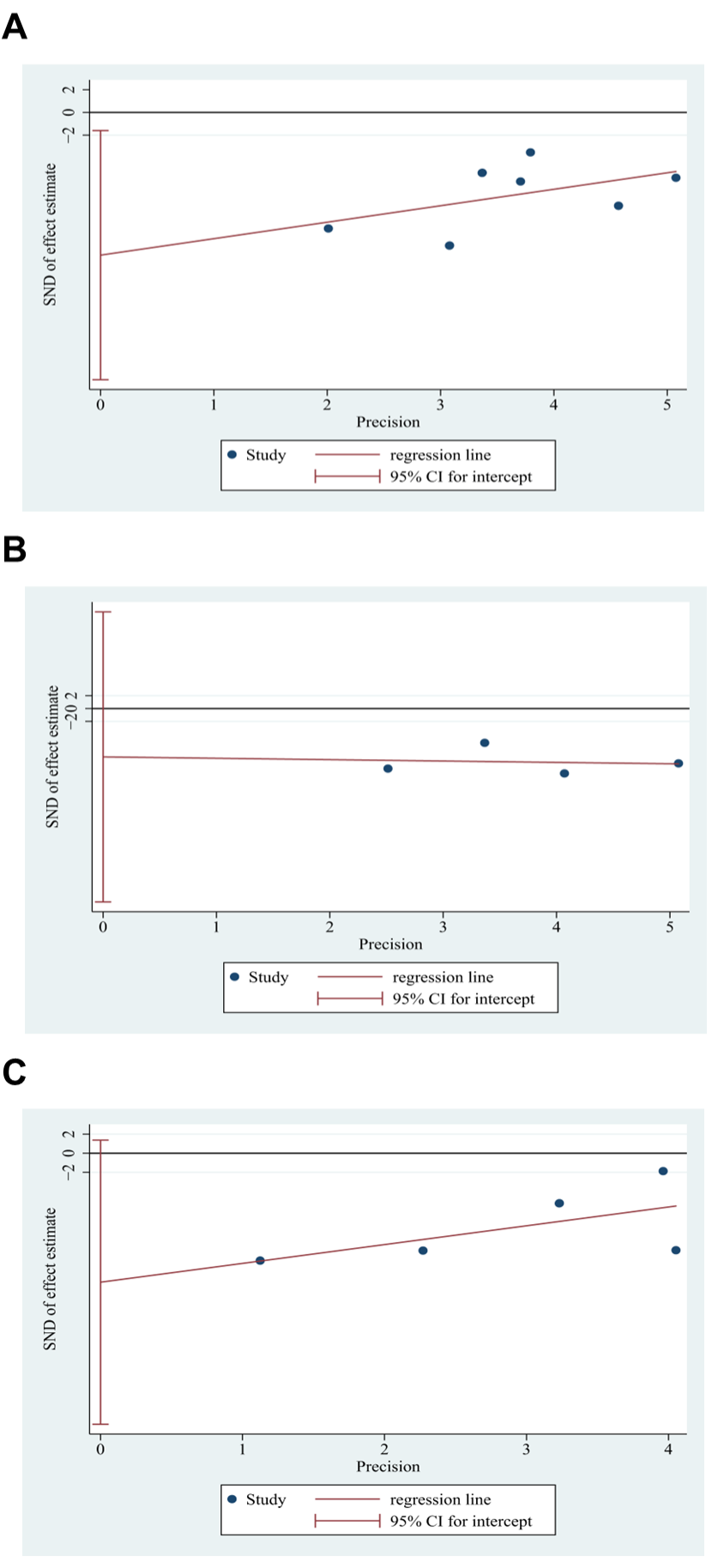


3. Egger's test for Fibrinogen levels(A), Hematocrit(B) and Plasma viscosity(C).


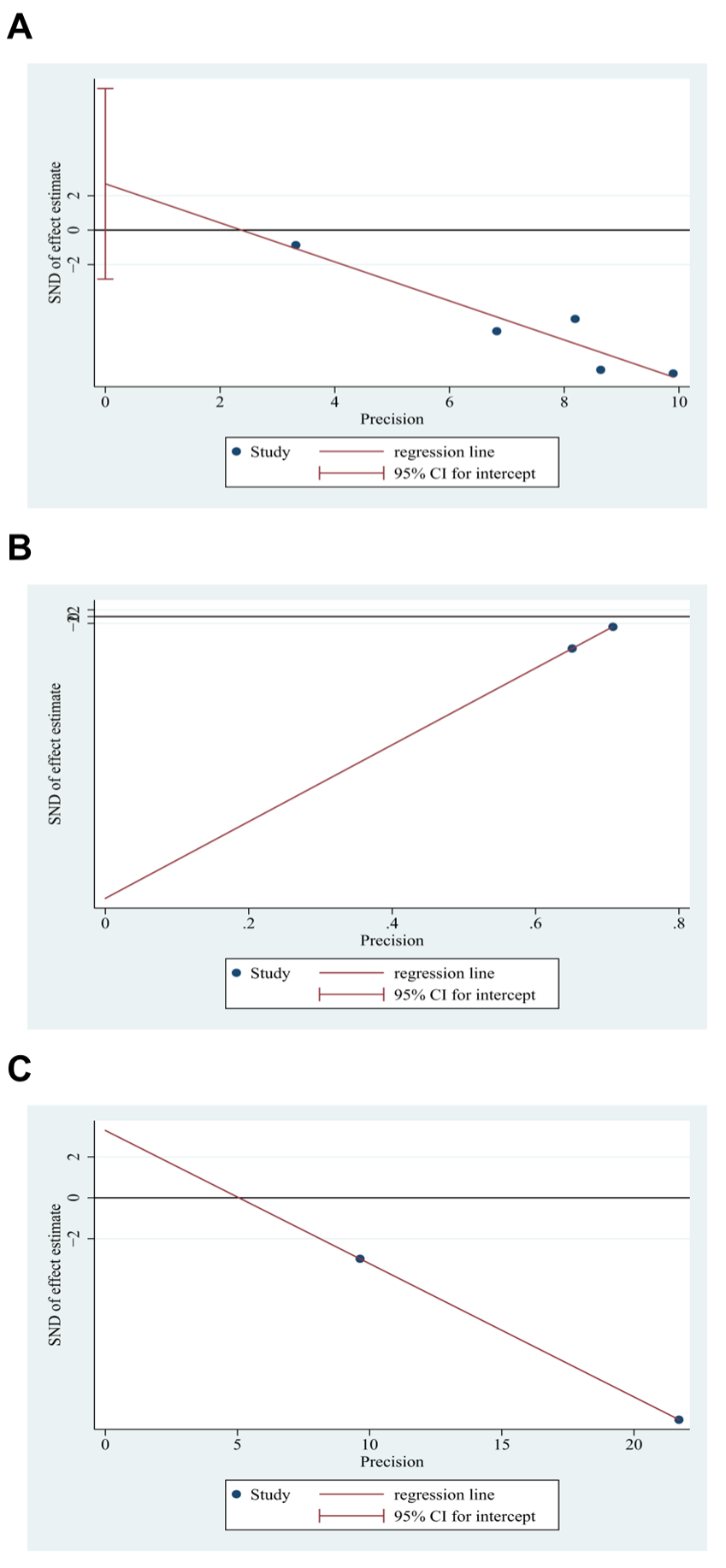


# Supplementary Table S4 The PRISMA checklist of this meta-analysis

| **Section and Topic** | **Item #** | **Checklist item** | **Location where item is reported** |
| --- | --- | --- | --- |
| **TITLE** | | |  |
| Title | 1 | Identify the report as a systematic review. |  |
| **ABSTRACT** | | |  |
| Abstract | 2 | See the PRISMA 2020 for Abstracts checklist. |  |
| **INTRODUCTION** | | |  |
| Rationale | 3 | Describe the rationale for the review in the context of existing knowledge. |  |
| Objectives | 4 | Provide an explicit statement of the objective(s) or question(s) the review addresses. |  |
| **METHODS** | | |  |
| Eligibility criteria | 5 | Specify the inclusion and exclusion criteria for the review and how studies were grouped for the syntheses. |  |
| Information sources | 6 | Specify all databases, registers, websites, organisations, reference lists and other sources searched or consulted to identify studies. Specify the date when each source was last searched or consulted. |  |
| Search strategy | 7 | Present the full search strategies for all databases, registers and websites, including any filters and limits used. | Supplementary TableS1 |
| . | 8 | Specify the methods used to decide whether a study met the inclusion criteria of the review, including how many reviewers screened each record and each report retrieved, whether they worked independently, and if applicable, details of automation tools used in the process. |  |
| Data collection process | 9 | Specify the methods used to collect data from reports, including how many reviewers collected data from each report, whether they worked independently, any processes for obtaining or confirming data from study investigators, and if applicable, details of automation tools used in the process. |  |
| Data items | 10a | List and define all outcomes for which data were sought. Specify whether all results that were compatible with each outcome domain in each study were sought (e.g. for all measures, time points, analyses), and if not, the methods used to decide which results to collect. |  |
|  | 10b | List and define all other variables for which data were sought (e.g. participant and intervention characteristics, funding sources). Describe any assumptions made about any missing or unclear information. |  |
| Study risk of bias assessment | 11 | Specify the methods used to assess risk of bias in the included studies, including details of the tool(s) used, how many reviewers assessed each study and whether they worked independently, and if applicable, details of automation tools used in the process. |  |
| Effect measures | 12 | Specify for each outcome the effect measure(s) (e.g. risk ratio, mean difference) used in the synthesis or presentation of results. |  |
| Synthesis methods | 13a | Describe the processes used to decide which studies were eligible for each synthesis (e.g. tabulating the study intervention characteristics and comparing against the planned groups for each synthesis (item #5)). |  |
|  | 13b | Describe any methods required to prepare the data for presentation or synthesis, such as handling of missing summary statistics, or data conversions. |  |
|  | 13c | Describe any methods used to tabulate or visually display results of individual studies and syntheses. |  |
|  | 13d | Describe any methods used to synthesize results and provide a rationale for the choice(s). If meta-analysis was performed, describe the model(s), method(s) to identify the presence and extent of statistical heterogeneity, and software package(s) used. |  |
|  | 13e | Describe any methods used to explore possible causes of heterogeneity among study results (e.g. subgroup analysis, meta-regression). |  |
|  | 13f | Describe any sensitivity analyses conducted to assess robustness of the synthesized results. |  |
| Reporting bias assessment | 14 | Describe any methods used to assess risk of bias due to missing results in a synthesis (arising from reporting biases). |  |
| Certainty assessment | 15 | Describe any methods used to assess certainty (or confidence) in the body of evidence for an outcome. |  |
| **RESULTS** | | |  |
| Study selection | 16a | Describe the results of the search and selection process, from the number of records identified in the search to the number of studies included in the review, ideally using a flow diagram. | Figure 1 |
|  | 16b | Cite studies that might appear to meet the inclusion criteria, but which were excluded, and explain why they were excluded. | Figure 1 |
| Study characteristics | 17 | Cite each included study and present its characteristics. | Table1 |
| Risk of bias in studies | 18 | Present assessments of risk of bias for each included study. | Figures 2A, 2B |
| Results of individual studies | 19 | For all outcomes, present, for each study: (a) summary statistics for each group (where appropriate) and (b) an effect estimate and its precision (e.g. confidence/credible interval), ideally using structured tables or plots. |  |
| Results of syntheses | 20a | For each synthesis, briefly summarise the characteristics and risk of bias among contributing studies. |  |
|  | 20b | Present results of all statistical syntheses conducted. If meta-analysis was done, present for each the summary estimate and its precision (e.g. confidence/credible interval) and measures of statistical heterogeneity. If comparing groups, describe the direction of the effect. |  |
|  | 20c | Present results of all investigations of possible causes of heterogeneity among study results. |  |
|  | 20d | Present results of all sensitivity analyses conducted to assess the robustness of the synthesized results. |  |
| Reporting biases | 21 | Present assessments of risk of bias due to missing results (arising from reporting biases) for each synthesis assessed. |  |
| Certainty of evidence | 22 | Present assessments of certainty (or confidence) in the body of evidence for each outcome assessed. | Table 3 |
| **DISCUSSION** | | |  |
| Discussion | 23a | Provide a general interpretation of the results in the context of other evidence. |  |
|  | 23b | Discuss any limitations of the evidence included in the review. |  |
|  | 23c | Discuss any limitations of the review processes used. |  |
|  | 23d | Discuss implications of the results for practice, policy, and future research. |  |
| **OTHER INFORMATION** | | |  |
| Registration and protocol | 24a | Provide registration information for the review, including register name and registration number, or state that the review was not registered. |  |
|  | 24b | Indicate where the review protocol can be accessed, or state that a protocol was not prepared. |  |
|  | 24c | Describe and explain any amendments to information provided at registration or in the protocol. |  |
| Support | 25 | Describe sources of financial or non-financial support for the review, and the role of the funders or sponsors in the review. |  |
| Competing interests | 26 | Declare any competing interests of review authors. |  |
| Availability of data, code and other materials | 27 | Report which of the following are publicly available and where they can be found: template data collection forms; data extracted from included studies; data used for all analyses; analytic code; any other materials used in the review. | Supplementary material |
